# Adaptive phenotypic modulations lead to therapy resistance in chronic myeloid leukemia cells

**DOI:** 10.1371/journal.pone.0229104

**Published:** 2020-02-27

**Authors:** Seda Baykal-Köse, Eda Acikgoz, Ahmet Sinan Yavuz, Öykü Gönül Geyik, Halil Ateş, Osman Uğur Sezerman, Güner Hayri Özsan, Zeynep Yüce

**Affiliations:** 1 Department of Medical Biology, Faculty of Medicine, Dokuz Eylul University, Izmir, Turkey; 2 Department of Histology and Embryology, Faculty of Medicine, Ege University, Izmir, Turkey; 3 Department of Histology and Embryology, Faculty of Medicine, Yuzuncu Yil University, Van, Turkey; 4 Department of Molecular Biology, Genetics and Bioengineering, Faculty of Engineering and Natural Sciences, Sabanci University, Istanbul, Turkey; 5 Department of Hematology, Faculty of Medicine, Dokuz Eylul University, Izmir, Turkey; 6 Department of Biostatistics and Medical Informatics, School of Medicine, Acibadem University, Istanbul, Turkey; Universita degli Studi di Firenze, ITALY

## Abstract

Tyrosine kinase inhibitor (TKI) resistance is a major problem in chronic myeloid leukemia (CML). We generated a TKI-resistant K562 sub-population, K562-IR, under selective imatinib-mesylate pressure. K562-IR cells are CD34^**-**^/CD38^**-**^, BCR-Abl-independent, proliferate slowly, highly adherent and form intact tumor spheroids. Loss of CD45 and other hematopoietic markers reveal these cells have diverged from their hematopoietic origin. CD34 negativity, high expression of E-cadherin and CD44; decreased levels of CD45 and β-catenin do not fully confer with the leukemic stem cell (LSC) phenotype. Expression analyses reveal that K562-IR cells differentially express tissue/organ development and differentiation genes. Our data suggest that the observed phenotypic shift is an adaptive process rendering cells under TKI stress to become oncogene independent. Cells develop transcriptional instability in search for a gene expression framework suitable for new environmental stresses, resulting in an adaptive phenotypic shift in which some cells partially display LSC-like properties. With leukemic/cancer stem cell targeted therapies underway, the difference between treating an entity and a spectrum of dynamic cellular states will have conclusive effects on the outcome.

## Introduction

Chronic myeloid leukemia (CML) is a clonal hematopoietic stem cell disease, clinically characterized by an increase in myeloid lineage cells at all stages of differentiation. The Philadelphia chromosome (derivative 22) derived from the t(9;22)(q34;q11) translocation, is the hallmark of the disease, transforming the hematopoietic stem cell (HSC) in to a leukemic stem cell (LSC) that gives rise to the disease. The translocation results in the fusion of the proto-oncogene ABL located on the long arm of chromosome 9, with the BCR gene on chromosome 22 [1]. The BCR-Abl oncoprotein possesses aberrant tyrosine kinase activity and provides survival signals to the malignant cells, which drive the disease in terms of cell proliferation and resistance to programmed cell death [2]. Despite being very resistant to conventional therapies, CML cells are sensitive to the blockage of the survival signal BCR-Abl provides. The introduction of imatinib-mesylate (IM) -the first tyrosine kinase inhibitor (TKI) used in the clinic- has redefined the management of CML. Patients with chronic phase disease, treated with imatinib achieve durable complete cytogenetic responses [3]. Nevertheless, some patients experience relapse and are resistant to imatinib [4]. Abl kinase domain mutations are the main culprit of TKI resistance, however, there is a subset of patients lacking these mutations and unresponsive to TKI treatment [5]. Amplification of the BCR-ABL oncogene, resulting in target molecules outnumbering intracellular concentrations of the TKI is another mechanism detected in unresponsive patients. Binding of imatinib to serum proteins and the role of drug influx and efflux proteins, limiting its intra-cellular bioavailability have also been implicated as resistance mechanisms [6]. Persistence of leukemic stem cells (LSCs) and a LSC-like phenotype based on BCR/Abl protein suppression have also been reported as TKI resistance mechanisms. [7] These defined mechanisms are far from covering all cases of TKI-unresponsive CML patients and in many cases the cause of resistance remains unknown [5]; suggesting yet unidentified mechanisms and additional routes involving epigenetic events or environmental factors. The persistence of LSCs despite long-term TKI-therapy is accepted to be the most important factor in leukemia progression related to TKI resistance. Recent studies have shown that changes in cell metabolism (oxygen/glucose shortage) suppresses BCR/Abl protein expression and favors the expansion of cells with a leukemia stem cell (LSC) phenotype. These LSC are refractory to imatinib mesylate and lead to TKİ resistant disease [7,8]

Phenotypic and functional heterogeneity arise among cancer cells within the same tumor as a consequence of the genetic mutations, environmental differences and reversible changes in cell properties. Recently, “phenotype switching” has been identified as an escape route for cancer cells [9]. By switching from a proliferative to an invasive state, cancer cells acquire resistance to therapeutics. Reversible phenotypic plasticity in tumor cells renders a proportion of cells to be more aggressive and resistant to therapy [10,11]. The most studied form of tumor cell plasticity is the epithelial-mesenchymal transition (EMT). EMT is a biological process that involves loss of cell polarity and cell-cell contact accompanied by the reorganization of the cytoskeleton, resulting in the conversion of epithelial cells to a characteristically invasive mesenchymal phenotype [12]. It has been shown that cancer cells can reside in various phenotypic states along the EMT spectrum, in which they can retain both epithelial and mesenchymal traits together, at varying degrees [11]. The hallmark of EMT is the loss of epithelial surface markers—mainly E-cadherin- and the acquisition of mesenchymal markers by an epigenetically mediated process [11,13,14].

EMT is a well-studied process involving the plasticity of tumor cells, yet cell plasticity is not limited to epithelial tumors. To understand the role of cellular plasticity in TKI-resistant CML cells, we generated a high dose imatinib-resistant K562 sub-population, K562-IR. We’ve shown that K562-IR cells are not only resistant to imatinib but also to 2^nd^, 3^rd^ generation TKIs and cytotoxic drugs. K562-IR cells are BCR-Abl-independent, no longer requiring it as a survival signal; characteristics comparable to LSCs. Yet, phenotypically K562-IR cells are not LCS, hence they are CD34 negative. They are highly adherent, proliferate slowly and show characteristics of a partially reprogrammed cell. Cell surface marker and protein analyses point towards an adaptive phenotypic shift that confers drug resistance. Hence, we show that therapies targeting driver mutations may not be sufficient to eliminate cancer cells due to an inherent plasticity which will let them undergo a phenotypic adaptation resulting in acquired resistance independent of secondary genetic events.

## Materials and methods

This study is approved by Dokuz Eylul University Non-surgical Ethics Comitee with the permit number: 2011/20-03.

### Cell lines

K562 (BCR-ABL-positive human CML cell line) was purchased from ATCC^®^. Cells were gradually exposed to increasing concentrations of imatinib (starting dose, 0,1μM; final dose, 10μM). 5μM is the maximum serum concentration (C_max_) reported in patients calculated after daily uptake of 400g Glivec [15]. The process generated an imatinib-resistant sub-population, K562-IR. Parental K562 cells were maintained in parallel cultures as control. Both cell lines were cultured in RPMI 1640 supplemented with 10% FBS, 1 unit/mL penicillin G, and 1 mg/mL streptomycin at 37°C in 5% CO^2^. K562-IR cells were continuously cultured in the presence of 10μM imatinib, “K562-IR w/o IM” indicates K562-IR cells grown in drug-free medium for four weeks.

### BCR-Abl kinase domain mutation, BCR-ABL gene expression and FISH analyses

RNA extraction and cDNA synthesis were performed by MN Nucleospin RNA II kit (Cat. No: 740955.50) and Fermentas M-MuLV Kit (#K1611) respectively. Primers were designed to generate four overlapping fragments in the BCR-Abl kinase domain (S1 Materials and Methods of S1 Table). PCR products were sequenced by Macrogen Inc., Korea. The sequencing results of each fragment were compared between K562 and K562-IR cell lines and with the NCBI BCR-Abl kinase domain default sequence using NCBI Blast online tool (https://blast.ncbi.nlm.nih.gov/Blast.cgi?PAGE_TYPE=BlastSearch). All analyses were done at three different time points (doi:10.6084/m9.figshare.c.4787100.v1). q-RT-PCR was performed using Roche Diagnostics TaqMan Master Kit (0453528001) with Roche Capillaire LightCycler v.4.0.0.23 software (Roche Diagnostics, Mannheim, Germany). FISH analysis was carried out with Cytocell Aquarius^®^ BCR/ABL Translocation Prob Dual Fusion (#LPH007) according to the manufacturer’s protocol. Three hundred randomly selected cells from K562 and K562-IR slides were counted and compared in terms of red (Abl), green (BCR) and fusion (yellow) signal number and amplification feature (disperse/cluster). Images were captured using NIKON Eclipse E600 epifluorescence microscope. Primers, BCR-ABL hydrolysis probe, and thermal profile are listed in S1 Materials and Methods of S2 Table.

### Treatment with tyrosine kinase inhibitors

K562 and K562-IR w/o IM cells were treated with imatinib, dasatinib, nilotinib, bosutinib and ponatinib with molarities corresponding to the highest plasma concentrations (C_max_) in patients [16–20] for 24, 48 and 72h, in 6-well plates. For high dose TKI treatment, both cell lines were treated with 10μM final concentration of each TKI in similar experimental settings. At each time point, cell viability was analyzed by trypan blue staining and Mitochondrial Membrane Potential/Annexin V Apoptosis Kit (Invitrogen, #V35116) according to the manufacturer’s instructions (S1 Results of S1 Fig).

### Cell viability analyses, proliferation and senescence assays

#### AnnexinV/Mitotracker apoptosis assay

Invitrogen Mitochondrial Membrane Potential/Annexin V Apoptosis Kit (#V35116) was used according to the manufacturer’s instructions. Results were interpreted as positive (living cells) by red fluorescence positivity and green fluorescence negativity which indicates living cells at the appropriate wavelengths via flow cytometry (BD FACSCanto II). (S1 Results of S1 Fig). WST-8 tetrazolium salt-based Cell Counting Kit-8 (Sigma-Aldrich, USA) was used as stated by the manufacturer for proliferation assays. Four 96-well plates were prepared for 0 h, 24h, 48h, 72h time points. Colorimetric analysis was performed with a microplate reader (BioTek Synergy HT) at 450nm absorbance. For senescence assays, 1x10^5^ cells were plated into 6-well plates. After 48h, 50nM doxorubicin was added to positive control wells. Assays were performed after 24h, using a SA-B-galactosidase based senescence assay kit (Cell Signaling, #9860) according to manufacturer’s instructions. All procedures were done in triplicate.

### Western blot and immunofluorescence staining

Protein lysates were prepared with RIPA buffer with 1mM PMSF and PhosSTOP phosphatase inhibitors (Sigma), quantified by Pierce BCA Protein Assay Kit (#23225) and analyzed by standard protocols. Antibodies are given in S1 Materials and Methods. For immunofluorescence staining, cells were plated on lysine-coated coverslips and processed by standard methods followed by incubation with primary antibodies against CD-44 (R&D BBA10) and E-cadherin (Abcam, ab760055) overnight at 4°C and stained with conjugated-secondary antibodies. Cells were visualized by fluorescent microscopy (Olympus BX-51 and the Olympus C-5050 digital test).

### Spheroid formation assay

1x10^4^ cells were plated per well in a 6-well plate pre-coated with 1ml of 3% Noble agar (w/v) (Difco Laboratories, Inc.; BD Diagnostic Systems, Detroit MI, USA) in serum-free RPMI 1640 and incubated at 37°C in 5% CO2 for 15 days. K562-IR cells are incubated with (10μM) and without imatinib. The number and diameter of colonies were photographed and counted every two days (Olympus BX-51, Germany) and the images of the representative fields were captured. Each sample was analyzed in triplicate.

### Flow cytometry assays for surface molecules

Cells were collected by centrifugation and fixed with 3.7% formaldehyde for 15 min at room temperature. After PBS washing steps, cells were stained with the conjugated-primary antibody or native primary antibody for 1h at room temperature in %5 BSA/PBS buffer. Cells were washed and stained with conjugated-secondary antibody for 30 min at room temperature in %5 BSA/PBS buffer. Following washing, cells are re-suspended in 500μl PBS and analyzed with BD FACS Calibur. Positive staining for each marker was determined by comparison with the only secondary antibody or appropriate isotype-matched of conjugated-primary antibodies as controls. Antibodies used are given in S1 Materials and Methods.

### Genome-wide gene expression profiling

K562 cells (K), K562-IR cells grown without imatinib (IR w/o IM), K562-IR in suspension grown with 10μM imatinib (IR w/ IM) and K562-IR adherent spindle-like cells grown with 10μM imatinib (S) were collected in triplicate and embedded in RNA-later solution (Invitrogen, Ambion, #AM7024) for further procedures. Transcriptome analyses were performed by AROS Applied Biotechnology, Denmark, with Illumina platform HumanHT-12 v.4 Expression BeadChip. Detailed data analyses are given in S1 Materials and Methods. Microarray validation procedures and primers are given in S1 Materials and Methods of S3 Table, respectively.

Detailed protocols for trans-differentiation experiments are given in S1 Materials and Methods.

## Results

### K652-IR cells are resistant to 1^st^ 2^nd^ and 3^rd^ generation TKIs and does not conform to known TKI resistance mechanisms

**Fig 1:**
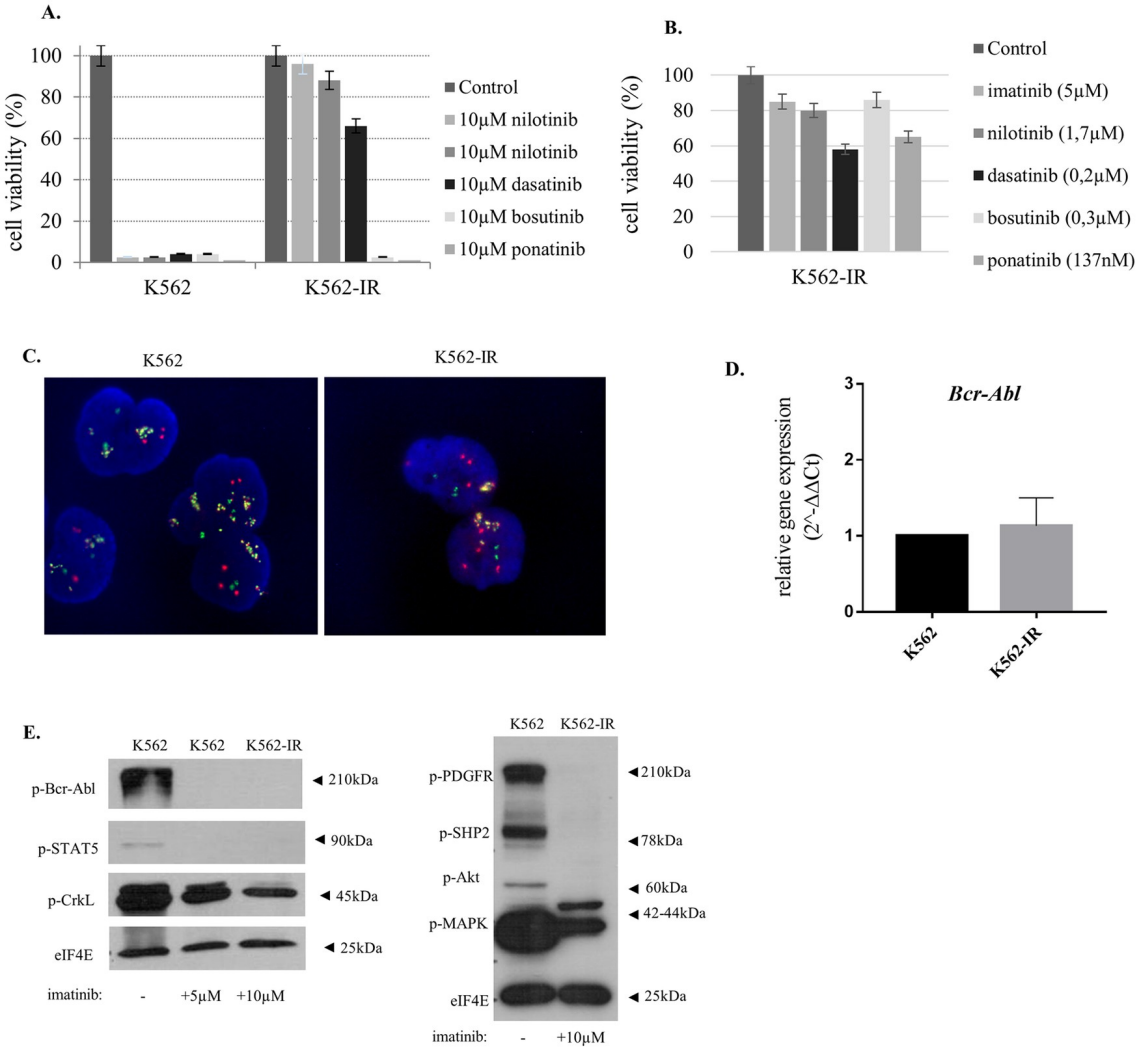
K562-IR cells are resistant to TKIs and do not conform to known TKI resistance mechanisms. A. Cell viability of K562 and K562-IR cell treated with 10μM TKIs B. Cell viability of K562-IR cells treated with C_max_ (daily clinical dose) concentrations. Flow cytometry analyses results were shown in the S1 Results of S1 Fig. Cells were treated with TKIs for 24, 48 and 72h followed by trypan blue staining and flow cytometry analyses for assessing cell viability. Only 72h results are presented. Viability was measured by counting live cells via hemocytometer after staining. Non-TKI treated cells were used for normalizing viability percentages. C. FISH analysis of K562 and K562-IR cell lines. Randomly selected 300 cells were analyzed for Bcr (green), Abl (red) and fusion (yellow) signals and amplification feature (cluster/disperse). The comparison of the two cell lines showed no significant difference. D. BCR-ABL q-RT-PCR gene expression levels revealed no significant difference between two cell lines (p>0.05) (n = 3 biological, 2 technical replicates in each). E. BCR-Abl and PDGFR pathway Western Blot analysis. Activation of proteins involved in BCR-Abl and PDGFR signaling pathway was studied using phospho-antibodies cocktails. eIF4E is loading control. BCR-Abl pathway was studied in cell lysates of K562, K562 grown in complete media supplemented with 5μM imatinib for 24h and K562-IR cells which were continuously incubated in complete media with 10μM imatinib. PDGFR pathway was studied using lysates of K562 as control and K562-IR cell which were continuously incubated in complete media with 10μM imatinib. Imatinib inhibits effectively BCR-Abl and PDGFR signaling in the K562-IR cells. Full-length blots and visualisation are presented in, S1 Raw image.

Established patient serum concentrations (C_max_) and 10μM were used in separate sets of experiments to assess TKI resistance. K562-IR cells are resistant to all therapeutics tested at molarities equivalent to C_max_ concentrations. Unlike C_max_ concentrations, extensive cell death was observed at 10μM concentrations of bosutinib and ponatinib (Fig 1A and 1B). It is of note that 10μM is 80-fold and 30-fold higher than the C_max_ in patients for ponatinib and bosutinib, respectively. Therefore, under a translational-to-the-clinic point of view, K562-IR cells were categorized as being resistant to bosutinib and ponatinib as well.

In the aim to identify underlying resistance mechanisms in K562-IR cells, we initially investigated the presence of ABL domain mutations (S1 Materials and Methods of S1 Table). Sequencing data revealed that no kinase domain mutations were present (n = 3 biological replicate). Both cell lines showed extensive oncogene amplification, albeit there was no difference between the two (Fig 1C). In addition, no difference in BCR-ABL mRNA expression levels was observed between K562 and K562-IR (Fig 1D). Drug influx-efflux mechanisms along with binding to serum proteins will render TKI intra-cellular bioavailability. Western blot with antibodies specific for phosphorylated BCR-Abl and its downstream molecules were performed in aim to show whether imatinib is available within the cell at sufficient quantities to inhibit BCR-Abl signaling. No difference in the inhibition of p-BCR-Abl, p-STAT5 and p-CrkL between K562 and K562-IR cells was observed; indicating imatinib is sufficiently active and functional in both cell lines. In the presence of imatinib, PDGFR signaling is also inhibited in both cell lines (Fig 1E). K562-IR cells are resistant to imatinib and other TKIs despite inhibited BCR-Abl and PDGFR signaling pathways, implying BCR-Abl signaling no longer provides a survival signal for these cells and the presence and function of imatinib are inconsequential.

### TKI-resistant K562-IR cells display phenotypic plasticity

**Fig 2:**
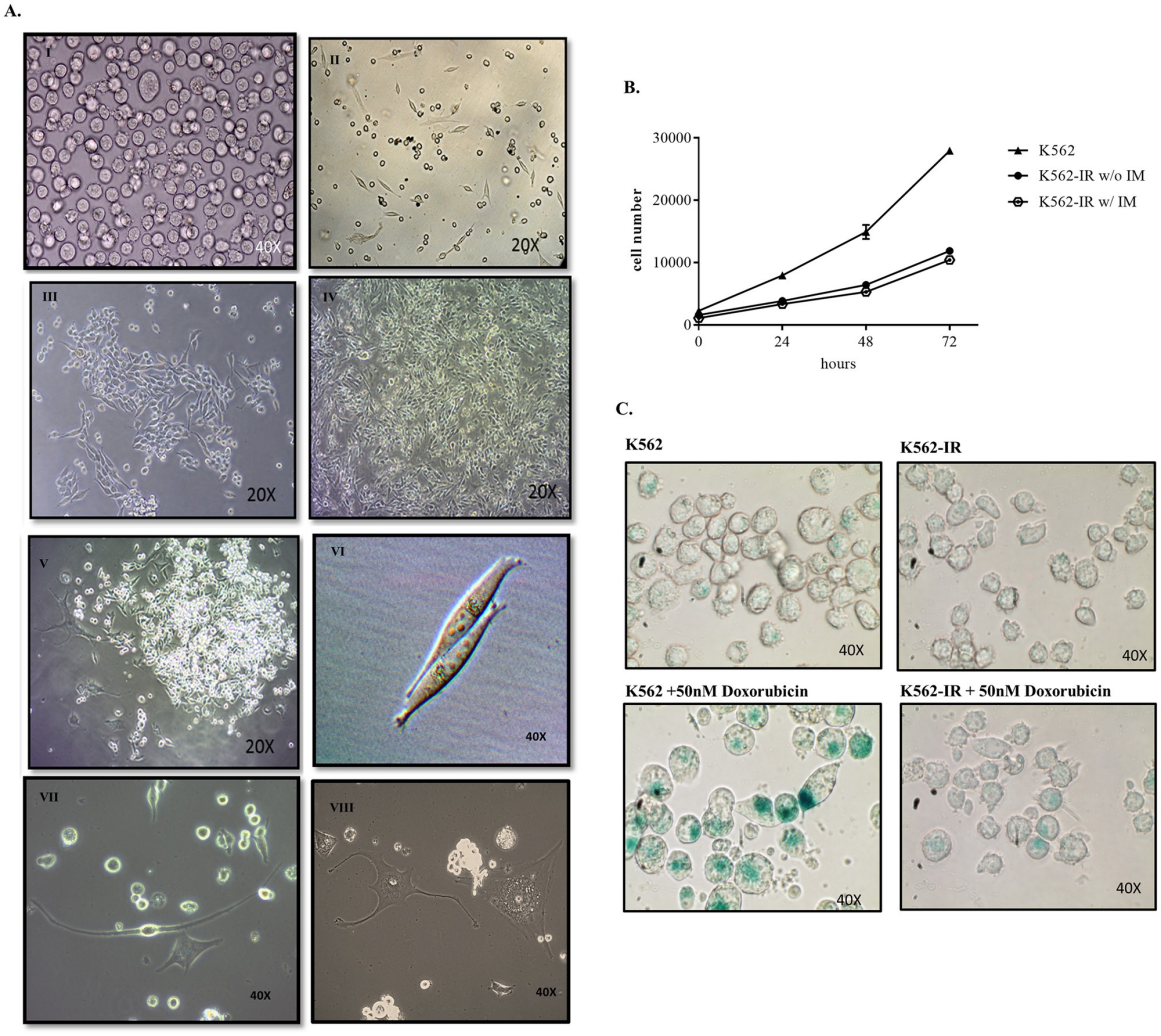
Cellular morphology and proliferative properties of K562-IR cells. A. K562 parental cells (I). K562-IR cells 24h day after of a new passage. A few adherent cells are observed. (II). The proliferation of adherent K562-IR cells (III). Monolayer of K562-IR cells after long-term culture and continuous removal of cells in suspension (IV). Adherent K562-IR cells forming colonies (V). Adherent K562-IR cells displaying different morphologies (VI-VIII) (Euromex inverted microscope, OX.2053 and DC5 optical imager). B. Comparison of the proliferation rates between K562, K562-IR w/o imatinib and K562-IR w/ imatinib. Cells at 24,48 and 72h time points using WST-8. The proliferation rate of K562-IR cells is approximately 4-fold slower than K562 at 72h (student t-test p≤0.01) (n = 3 biological replicates) C. ß-galactosidase senescence assay of K562 and K562-IR growing in media with imatinib. 50nm doxorubicin was added for 24h as positive control (upper right-lower right). The presence of imatinib does not trigger senescence in K562-IR cells. While doxorubicin induces senescence in K562 cells, K562-IR cells are resistant to this effect. (Euromex inverted microscope, OX.2053 and DC5 optical imager).

K562-IR cells have the potential to adhere and form monolayers (Fig 2A). Wild-type K562 cells normally grow in suspension. After successive clonal selection for imatinib resistance, cells were observed to adhere to the flask surface and gain a spindle-like shape. Although K562-IR cells are morphologically indistinguishable from the parental K562 cell line when grown in suspension; when grown in tissue culture flasks and left undisturbed they were observed to form foci and monolayer sheets. In sequential splits of K562-IR, 15–50% percent of cells growing in suspension developed adherent, fibroid morphology, implying the capacity of a dynamic and reversible phenotype switch. To verify that these adherent cells originated from K562, authentication analyses were performed (S1 Results of S2A, S2B, S2C Fig).

### TKI-resistant K562-IR cells proliferate slower than their parental counterparts and are resistant to doxorubicin-induced senescence

Another prominent observation is that the K562-IR cells proliferate 4-fold slower than K562, independent of the presence of imatinib (Fig 2B). To exclude the possibility that these cells are prone to senescence or that the presence of imatinib in growth media may be inducing senescence, SA-B-galactosidase activity was tested in both cell lines. Without induction, senescent cells were not observed in neither K562 nor K562-IR cells. Following doxorubicin induction, K562 cells entered senescence whereas K562-IR cells were not affected (Fig 2C). K562-IR cells are resistant to doxorubicin-induced senescence implying resistance to cytotoxic drugs as well as TKIs.

### TKI-resistant K562-IR cells form tumor spheroids, express high levels of E-cadherin, caveolin-1, CD44 and decreased β-catenin

**Fig 3:**
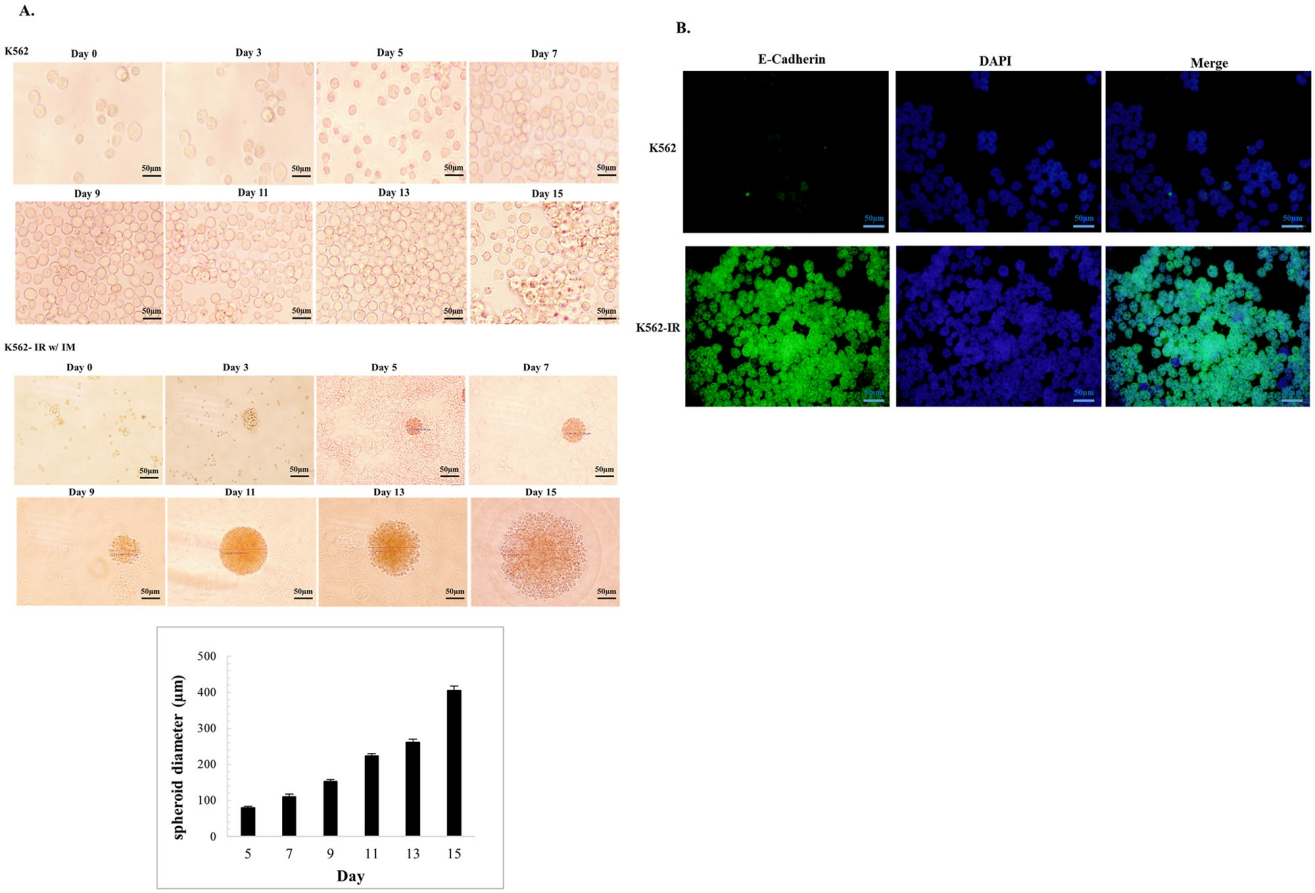
K562-IR cells form intact tumor spheroids and express E-cadherin. A. Spheroid formation assay of K562 and K562-IR w/ IM (10μM) cells in agarose culture for 15 days. K562 cells do not form tumor spheroids whereas K562-IR cells show extensive spheroid formation (n = 3 biological replicate). Spheroid diameter measurements of K562-IR cells were performed every two days using an Olympus BX-51 microscope. B. E-cadherin immunofluorescence staining of K562 (upper row) and K562-IR (lower row) cells (Olympus BX-51 and the Olympus C-5050 digital test). K562-IR cells display high expression levels of E-cadherin.

K562, K562-IR, and K562-IR grown w/o imatinib were incubated in soft agar culture medium for two weeks. Multicellular tumor spheroid formation was observed only in the K562-IR cells, independently of the absence or the presence of IM in culture medium, with comparible diameters. This observation suggests that imatinib is not involved in spheroid formation (Fig 3A and S1 Results of S6 Fig). E-cadherin is the major adhesion molecule mediating tight cell-cell interaction and has been correlated with compact spheroid formation [21]. E-cadherin is not expressed in the parental K562 cell line as expected from hematopoietic cells. On the contrary, E-cadherin expression was found to be remarkably high in resistant K562-IR cells (Fig 3B).

**Fig 4:**
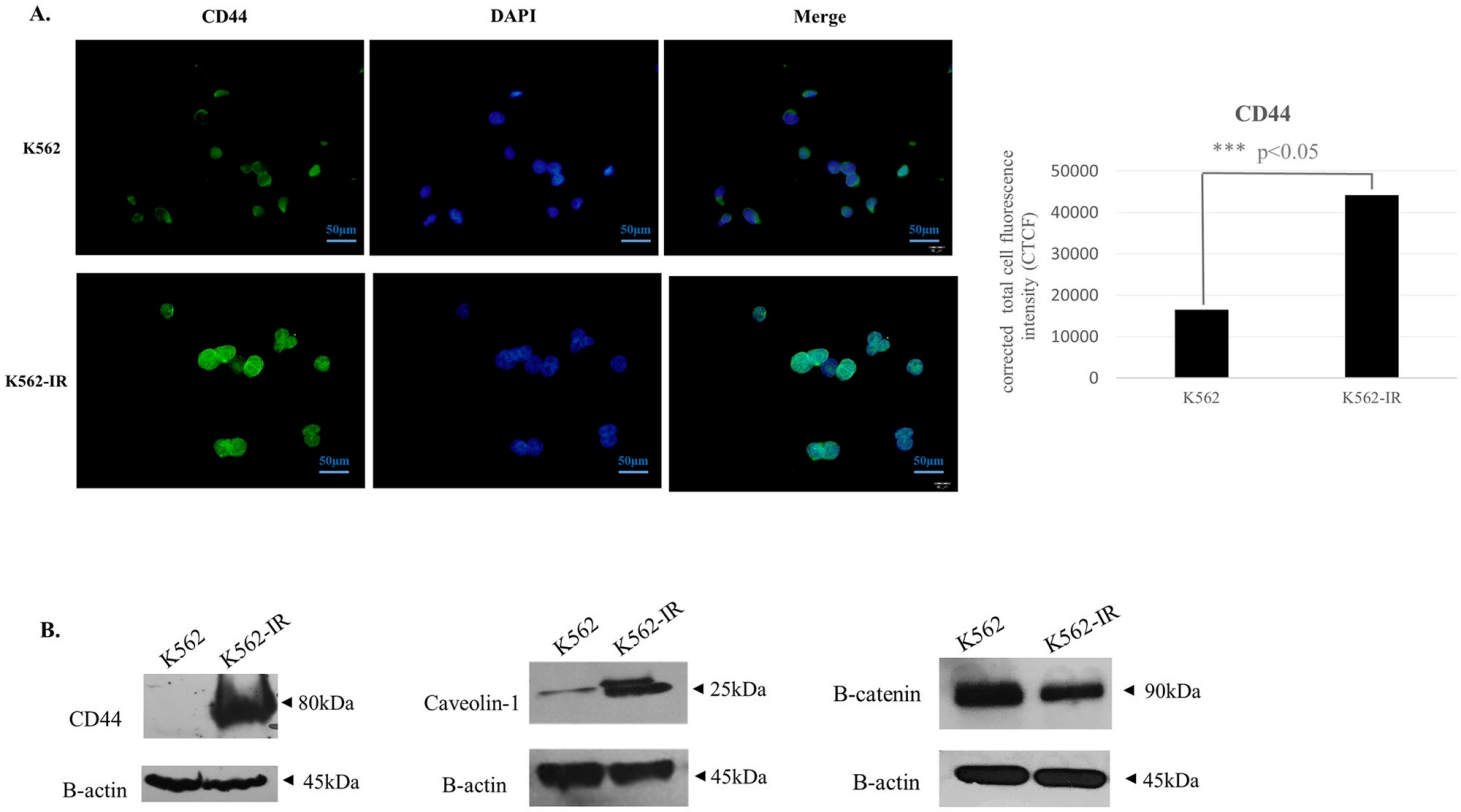
Protein expressions of CD44, Caveolin-1, and B-catenin in K562 and K562-IR cells. A. CD44 immunofluorescence staining. DAPI was used for nuclear visualization. Total corrected cellular fluorescence (TCCF) intensity of CD44 immunofluorescence images (right). (TCCF = Integrated Density–area of selected cell X mean fluorescence of background readings). Measurements were performed using the ImageJ software ^40^. K562-IR cells express CD44 protein higher than K562 (student t-test p ≤ 0.001) B. Western blot analyses of CD44, Caveolin-1, and B-catenin show that these proteins are highly expressed in K562-IR. B-catenin expression has decreased in K562-IR cells when compared to K562. B-actin and eIF4E were used as loading controls. (n = 3 biological replicates). Full-length blots and visualisation are presented in S1 Raw image.

Recent studies have reported cross-talk between E-cadherin, Caveolin-1, β-catenin and TGF-β signaling [22–26]. Caveolin-1 protein is highly expressed in the K562-IR cell line when compared to K562. In light of studies reporting caveolin-1 interaction with E-cadherin, the increase of E-cadherin expression in concomitance with that of Caveolin-1 in the K562-IR cell line may suggest that these two proteins work in concert. β-catenin expression is decreased in K652-IR cells (Fig 4B). The decrease in β-catenin supports the slow proliferation rate observed in K562-IR cells. E-cadherin sequesters cytoplasmic β-catenin which is necessary for the transcription of genes of the proliferative Wnt signaling pathway. On the other hand, β-catenin is a crucial molecule in HSC and LSC maintenance [27] and a decreased expression does not support a stem-like phenotype. Another critical molecule in the regulation and maintenance of stem-like features is CD44, which has been shown to have a role in the localization of LSCs, and its suppression results in their elimination [28–31]. The K562-IR cell line expresses high levels of CD44, as shown by immunofluorescence and western blot assays (Fig 4A and 4B).

### TKI-resistant K562-IR cells have an altered surface marker profile

**Fig 5:**
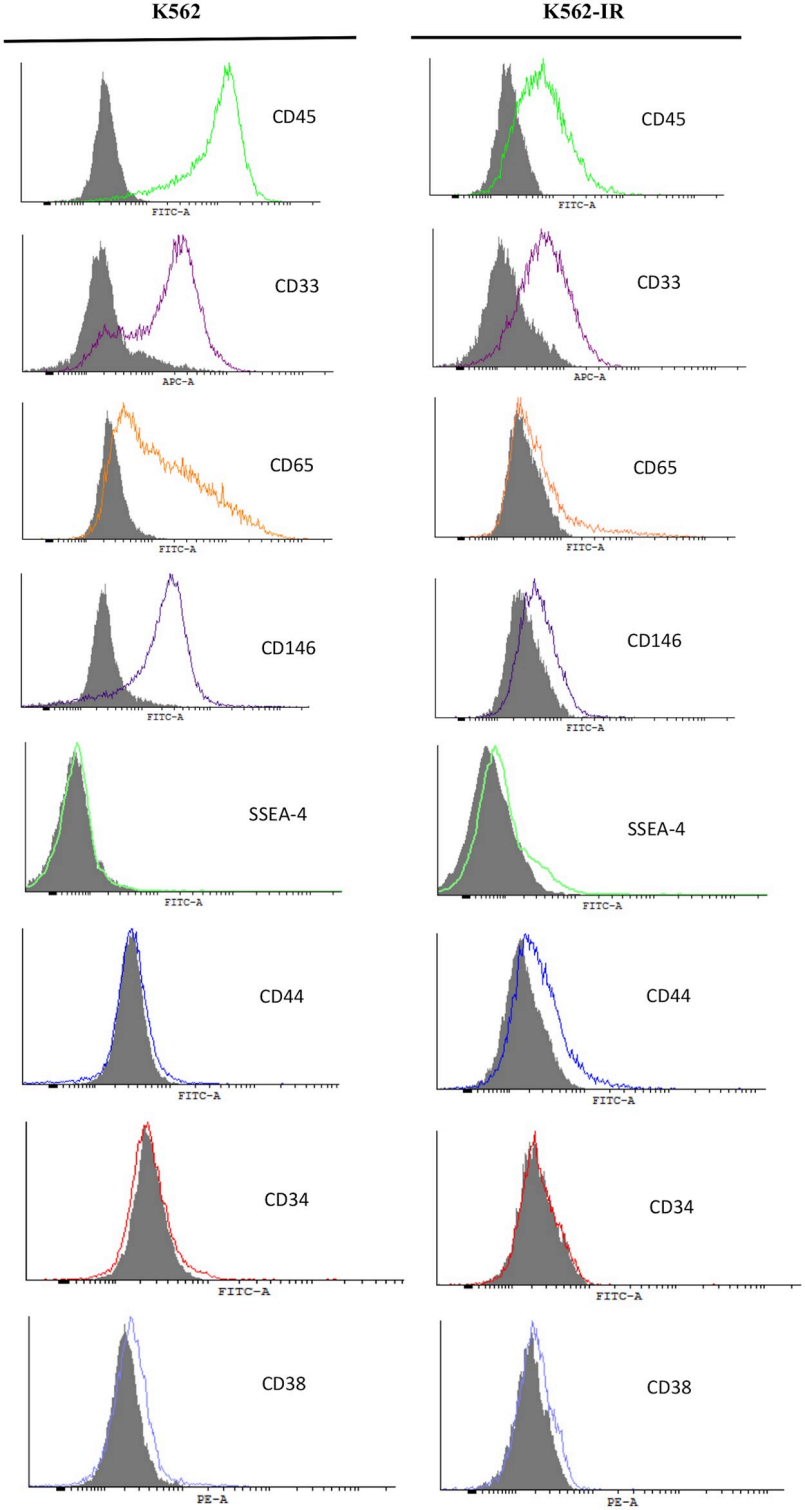
Flow cytometry of cell surface markers of K562 and K562-IR cells. CD45, CD33, CD65, CD146 hematologic marker expressions are reduced in K562-IR cells. CD44 and SSEA-4 have increased expression while no difference was observed in CD34 and CD38. Grey area in the histograms is background signal from isotype control. (n = 3 biological replicates).

Tra-1-60 and Tra-1-81 were slightly positive in both ancestral and resistant cell populations with no difference in expression between the two groups. (S1 Results of S5 Fig) SSEA-4 is negative in ancestral K562 cells, while K562-IR cells display a low level of positivity (Fig 5). The expression of CD45 (leukocyte common antigen-LCA, the general marker for hematopoietic cells) is significantly reduced in resistant cells; suggesting that K562-IR cells are gradually drifting away from their hematological origin. Cell surface expression of CD33, CD146, and CD65 was also decreased in K562-IR.

CD34 and CD38 are markers for both HSC and LSCs. K562 and K562-IR cells are negative for CD38 in repeated experiments. CD34 expression was either negative or displayed low positivity (1–4%) and comparable in both cell lines (Fig 5).

### TKI-resistant K562-IR cells do not differentiate into other mesenchymal lineages

Both surface protein expressions and cellular morphology have shown that K562-IR cells phenotypically shifted away from their hematological origins. We performed functional essays to test MSC-like features. K562 and K562-IR cells were tested for differentiation to adipocytes and osteoblasts when grown in inductive media. Neither cell line displayed adipocyte- or osteocyte-like features (S1 Results of S3A-S3B-S3C Fig). K562-IR cells did not display a potential to differentiate to other mesenchymal lineages in our experiments.

### Gene expression profiling indicates K562-IR cells differentially express tissue/organ development and differentiation genes

**Fig 6:**
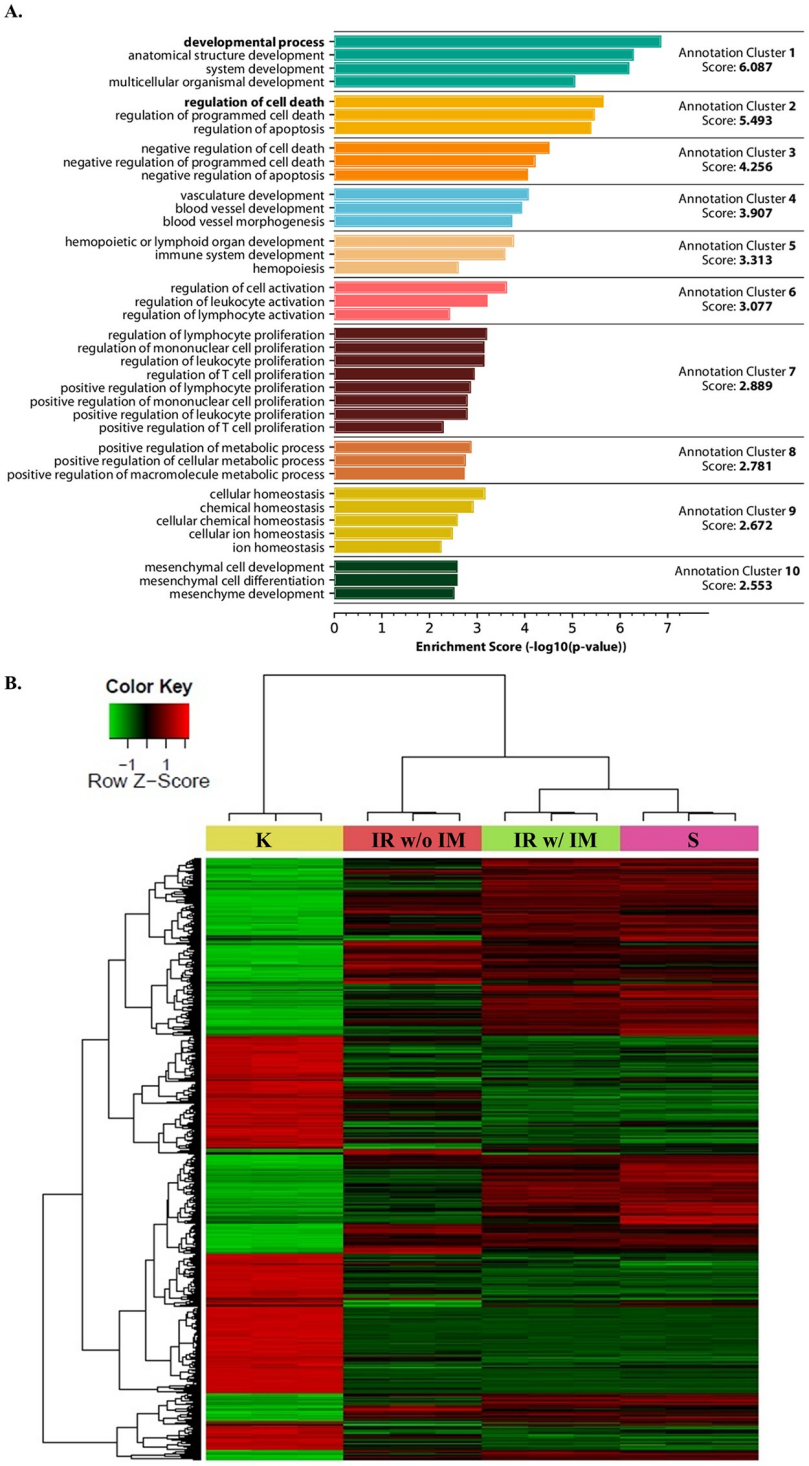
Gene Ontology and heatmap analysis of transcriptome data. A. Functional annotation cluster analysis based on Gene Ontology (GO) Biological Process (BP) terms. Analysis was performed using DAVID 6.7 database for differentially expressed genes (FDR < 0.05 and fold change > 2 or < 2) of IR w/ IM vs. K comparison. B. Heatmap generated from triplicate microarray analyses of differentially expressed genes considering a FDR adjusted p-value less than 0.05 as statistically significantly changed. Abbreviations: “K”, K562 cells incubated in complete media; “IR w/o IM”, floating K562-IR cells in suspension incubated in complete media without imatinib for four weeks; “IR w/ IM”, floating K562-IR cells in suspension continuously incubated in complete media with 10μM imatinib; “S”, spindle-shaped K562-IR cells continuously incubated in complete media with 10μM imatinib.

Three groups of resistant K562-IR cells (IR w/ IM, with imatinib; IR w/o IM, without imatinib/suspension cells; and S, IR with imatinib/adherent spindle-shaped cells) were compared to parental the K562 cell line (K). The most common biological processes relative to gene ontology taken into consideration for enrichment analysis are associated with development, differentiation, and morphology. According to functional annotation analysis, three of the first five clusters are related to development/differentiation biological processes; in addition, the tenth cluster is enriched in mesenchymal cell development (Fig 6A). 462 and 310 genes involved in developmental and differentiation processes, respectively, are differentially expressed in the K562-IR cells compared to ancestral K562 (S1 Results of S4 Fig) The gene expression profiles of the three groups of resistant cells are more related to each other than to the ancestral K562 cells as seen in heatmap analysis (Fig 6B). Interestingly, the gene expression profile of the IR w/o IM group does not extensively overlap with the other two resistant cell groups. In addition, these cells have not converted back to the expression profile of K562 cells. These findings reveal the acquisition of a new transcriptional background reflecting an altered phenotype for the resistant cells. This altered phenotype does not overlap with LSC/CSC features, but rather reflects a loss of identity or an intermediate metamorphic state.

## Discussion

The BCR-Abl oncoprotein provides a survival signal for CML cells. Unlike conventional chemotherapy approaches, the success of TKIs in the treatment of CML lies in triggering cell death by blocking BCR-Abl signaling. Nevertheless, resistance to TKIs still remains a challenge in a subgroup of patients. Known resistance mechanisms do not cover all cases and yet unidentified mechanisms and additional routes involving epigenetic events have been suggested as a cause where known mechanisms have been excluded.

We treated K562 cells with increasing doses of imatinib, resulting in the selection of a high dose TKI-resistant sub-population of K562 cells—K562-IR. The K562-IR cell line proliferates slower than the parental cell line, is capable to adhere and grow as a monolayer, and shows characteristics of a partially reprogrammed cell. Cell surface marker and protein analyses point towards a phenotypic shift towards a non-hematopoietic cell, implying an adaptation process in the presence of BCR-Abl inhibition. K562-IR cells are highly resistant to TKIs. Sequence analysis of resistant and ancestral K562 cells for the BCR-Abl domain region did not reveal any base changes. Similarly, there was no difference between the resistant and sensitive cells in terms of BCR-ABL gene amplification. The endpoint of other defined TKI resistance mechanisms can be generalized as the inability of TKIs to reach intracellular therapeutic concentrations. Intracellular imatinib concentrations were tested indirectly by comparing inhibition of BCR-Abl signaling. Autophosphorylation of BCR-Abl and downstream proteins were reduced following imatinib treatment in both K562 and K562-IR cells. Although imatinib was shown to be effective and sufficient in inhibiting BCR-Abl signaling in K562-IR cells, cell death is not observed, suggesting that BCR-Abl kinase activity is no longer a survival signal. It is of note that our transcriptome analysis showed an increase in the expression of MDR (multidrug resistance) related ABC family genes in K562-IR cells. The presence of any chemical stress factor in the environment will lead to the increased expression of these transporter genes. Based on two observations, we did not interpret this as a mechanism of resistance: 1) Transcriptome data reveals a statistically significant increase in ABCC3, ABCB1, and ABCA1 gene expressions when imatinib is present, whereas when the stress factor (imatinib) is removed, gene expression relatively decreases as expected; 2) BCR-Abl downstream protein phosphorylation reveals that intracellular imatinib concentrations are sufficient to inhibit BCR-Abl signaling indicating that TKI are active and thereby that TKI resistance is not associated with increased drug efflux.

The most important observation is the phenotypic plasticity of K562-IR cells. The parental K562 cells are of hematopoietic origin, round-shaped and grow in suspension. During clonal selection under selective imatinib pressure, adherent spindle-shaped cells capable of growing as a monolayer emerged. 168 genes involved in adhesional processes are differentially expressed in the K562-IR cells compared to ancestral K562 (S1 Results of S4 Fig). When K562-IR cells suspended in culture media above the adherent cells are removed and transferred to new flasks, a subset of cells repeatedly adhere to the culture vessel while maintaining a suspended population; suggesting that the phenotypic plasticity observed in TKI-resistant K562-IR cells is a dynamic and reversible phenomenon.

K562-IR cells proliferate slower than parental K562 cells. To exclude the possibilities that the presence of imatinib in growth media may induce senescence or that these cells are inherently prone to senescence, we tested for SA-B-galactosidase in both cell lines. In the absence of inductive treatment, no senescent cells in neither the ancestral K562 cell line nor in the K562-IR cells (with or w/o imatinib) were observed. K562 cells enter senescence after treatment with doxorubicin, in the positive control groups; whereas K562-IR cells are not affected by and resistant to senescence induced by doxorubicin -a conventional therapeutic agent- implying a resistance to cytotoxic drugs as well as TKIs. Slowly proliferating cells in cancer cell populations are frequently associated with cancer/leukemic stem cells (CSCs/LSCs). CSCs/LSCs are defined as a subgroup cell with the capacity for self-renewal and generating all cancer cell lineages of the original tumor. LSCs/CSCs are crucial players in tumor relapse and therapy resistance with important clinical implications. Many studies have demonstrated that CML is maintained by LSCs, mirroring the hierarchically-structured normal hematopoietic system maintained by self-renewing HSCs [32]. CML LSCs do not depend on BCR-Abl signaling for their survival and are free from oncogene addiction [33]; thus are able to survive under prolonged TKI treatment.

Considering the morphological and transcriptional differences of the K562-IR cells, we examined their behavior in a semi-solid environment to investigate colony forming capacity. The capability to form spheroids in vitro is regarded as a convenient surrogate to evaluate tumor-initiating potential [34]. In multicellular tumor spheroid assays, while K562-IR cells form smooth-walled, tightly-packed dense spheroid structures, this type of spheroid formation is not detected in K562 ancestral cells. Xin et al. emphasized in a study that the well-established multi-drug-resistant cell line K562/A02, formed compact and “relatively” large spheroids compared to K562 cells in serum free medium containing EGF and bFGF [35]. In this study, spheroid formation of K562 cells could be related to the effect of growth factors, although no significant compact spheroid structures were observed. Previous studies have also reported that growth factors have an effect on mitogenic potential, differentiation capacity and spheroid formation efficiency [36–38]. In addition, Puissant et al. reports that an imatinib-resistant and highly adherent K562 cells (IM-R Adh) can produce spheroid, whereas imatinib-sensitive (IM-S) counterparts cannot [39]. The spheroid formation is associated with E-cadherin expression, an epithelial-derived surface-binding molecule of the cadherin superfamily. K562-IR cells express high levels of E-cadherin, both in their suspension and adherent forms. The fact that K562-IR cells express an epithelial marker suggests that these cells have undergone a significant phenotypic shift via transcriptional/epigenetic changes under stress conditions. E-cadherin is a critical molecule in tumor cell plasticity defined in the context of EMT [11–14]. In normal cells, E-cadherin sequesters cytoplasmic β-catenin which is necessary for the transcription of genes of the proliferative Wnt signaling pathway. Loss of E-cadherin and the accumulation of β-catenin are observed in EMT; whereas the reverse is true for mesenchymal-epithelial transition (MET) [26]. EMT/MET processes as defined in literature are not comprehensively relevant to these cells that are derived from hematopoietic tissue. In addition, activated Wnt/β-catenin signaling has been shown to be essential in HSCs and CML LSCs [27,40]. Although K562-IR cells display spheroid formation associated with CSCs; they also have decreased β-catenin expression that does not corroborate with LSC features. K562-IR cells are not epithelial cells, they are able to grow as adherent cells but are morphologically indistinguishable from K562 cells when in suspension. Recent investigations have argued that EMT-MET is not a binary process, rather cells can attain stable hybrid epithelial/mesenchymal phenotypes [41]. Hybrid epithelial/mesenchymal phenotypes may not merely be steps that epithelial cells undergo during embryogenesis and metastasis, but also adaptive states that a cell reverts to under environmental triggers, regardless of its original lineage.

To further characterize K562-IR cells, flow cytometry studies were performed with antibodies against hematopoietic cell and stem cell, mesenchymal stem cell, and pluripotency markers. CD34 and CD38 are basic hematopoietic stem cells and LSC markers. Both K562 and K562-IR cells were found to be CD34^-^/CD38^-^. The CD34^-^**/**CD38^-^ profile does not coincide with “stemness” and reflects a more differentiated phenotype. Pluripotency markers Tra-1-60 and Tra-1-81 surface proteins displayed low-level positivity in both K562 and K562-IR cell populations with no difference in expression between the two groups. SSEA-4, a molecule synthesized in pluripotent cells is negative in K562 cells, while a low level of positivity is observed in K562-IR cells. CD45 an important marker for cells of hematopoietic origin is significantly reduced in K562-IR, supporting evidence that they have drifted away from their hematological origins. In line with these observations, CD146 expression -which is exclusively expressed in myeloid cells-, is decreased in K562-IR.

CD44 is a widely used CSC marker in solid tumors and also recognized as a regulator of LSC maintenance in the hematopoietic stem cell niche [42]. CD44 protein was expressed at high levels in K562-IR cells. The defined Ph^+^ CD34^+^ LSC compartment in CML is proposed to be BCR-Abl-independent and resistant to TKI treatment. K562-IR cells are negative for CD34 but positive for another stem cell marker CD44; and as CML LSCs, they are BCR-Abl independent. They express both epithelial and mesenchymal markers associated with cellular plasticity. Our data suggest K562-IR cells display some characteristics of LSCs but do not conform in terms of phenotypic markers of the CML LSC phenotype described in literature. When functionally tested for MSC properties, K562-IR cells did not convert to adipocytes or osteocytes in trans-differentiation experiments. Our results reveal that these cells are partially reprogrammed cells that have lost their identity and reside in a metamorphic state to maintain self-preservation.

K562 cells have been extensively used as an in vitro model system for studying the differentiation along the erythroid and megakaryocytic-monocytic lineages. There are numerous studies in literature that have reported phenotypic changes in K562 cells following treatment with different compounds to induce differentiation. Treatment with phorbol esters has been shown to induce differentiation along a megakaryocytic-monocytic lineage whereas treatment with hemin, 5-azacytidin, l-/3-D-arabinofuranosylcytosine, daunomycin or herbimycin A can induce differentiation along an erythroid lineage [43–49]. Induced differentiation of K562 with various compunds leads to a slower proliferation rate and alterations in adhesion properties of these cells. However in all of these studies K562 cell have differentiated in to cells of hematological origin and have not been reported to express epithelial markers. In addition, these induced differentiation studies do have not resulted in K562 cells growing as monolayers.

Two different studies support our observations. Puissant et al. reported an adherent subpopulation of K562 selected under imatinib pressure, with a CD34^+^/CD44^high^/CD24^low^ phenotype [39]. They reported increased transmigration and invasion in this CML cell subpopulation through overexpression of the αVβ3 integrin receptor leading to FAK/Akt pathway activation. Chorzalska et al. also reported that prolonged exposure to imatinib in culture resulted in adherent K562 cells. They demonstrated the upregulation of pluripotency markers and adhesion receptors in TKI-resistant adherent CML cells [50]. Both groups’ data and ours support prolonged imatinib exposure results in cellular plasticity and an adherent phenotype acquisition. Nevertheless, there are reported differences in phenotypic markers and gene expression. We suggest that the observed differences in marker and gene expression are a result of transcriptional instability in search of a stable gene expression framework, suitable for new environmental stresses. In another report, CML cells incubated in low oxygen, were shown to completely suppress the BCR/Abl protein [8]. Cells selected in low oxygen display LSC like properties and are resistant to imatinib because their molecular target is suppressed. However, we have shown that K562-IR cells continue to express BCR-Abl protein, and do not display LSC surface markers. New environmental stressors result in selection pressures favoring divergence from the ancestral state. Cells capable of losing identifiable qualities and de-differentiate to a more primitive state may display higher fitness in challenging environments. Partially reprogrammed cells have been a focus of interest in recent years. Reprogramming is a gradual process [51]. The intermediate states during the generation of pluripotent cells have overlapping features regardless of cellular origin. Genome-wide expression analyses have shown that partially reprogrammed cell lines derived from B cells and fibroblasts are more similar to each other than to their cells of origin. In addition, inhibition of differentiation-associated pathways is crucial for inducing pluripotency [51,52]. An intermediate/ primitive metamorphic cellular state may be a common feature of phenotypic switching, whether induced in a lab or emerged naturally as a response to environmental stress.

**Fig 7:**
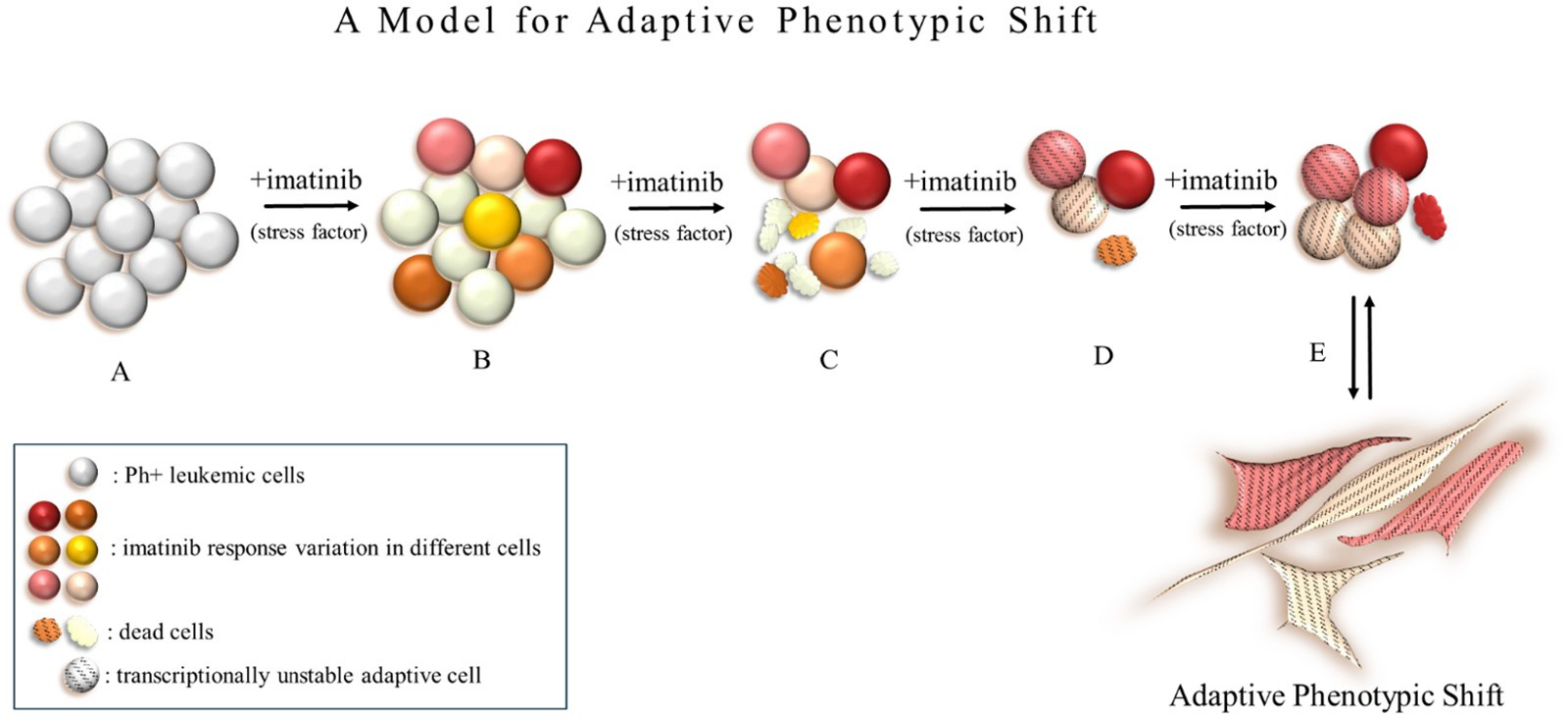
Adaptive phenotypic shift model as a TKI-resistance mechanism in CML cells. (A) Ph+ leukemic cell population. (B) Imatinib susceptibility in Ph+ cells is not homogenous and is shown in different colors. (C) The most susceptible cells will die off immediately while imatinib response of other cells in the same population will differ and cell death will occur over time. (D) Cells with the ability to acquire a transcriptional instable state in search of an adaptive landscape that confers higher fitness, some cells will die during this while others survive by breaking through the BCR-Abl-driven life cycle. (E) Cells exhibiting adaptive phenotypic plasticity will survive under selective conditions, may dominate the population or stay dormant in a primitive adherent state within the niche until the environmental stressor (imatinib) is removed.

Adaptation by phenotypic plasticity is achieved by transcriptional instability; defined as the search for alterations in the expression of necessary genes required to change the epigenetic landscape of the cell. This landscape is a dynamic structure and it is plausible that the transcriptional infrastructure may be instable. In the context of imatinib resistance, adaptive flexibility offers a unique resistance model. Due to fluctuations in mRNA and protein expressions of each cell, a variable sensitivity exists in CML cells to identical active intracellular imatinib concentrations. Transcriptional instability as a stress response may result in acquired transcriptional flexibility allowing for a BCR-Abl independent phenotype to emerge. Targeted/conventional therapies may actually promote phenotypic switching in neoplastic cells, allowing them to acquire resistance. An adaptive phenotypic shift (APS) to a relatively primitive “no-name” phenotype will confer upon cells BCR-Abl independency (Fig 7). Cells displaying APS will be able to reside dormant in the bone marrow niche with the capacity to revert back to its original phenotype once the stressor is removed or with the accumulation of additional mutations. Cells undergone APS will display different transcriptional frameworks; some partially, others fully compatible with the LSC/CSCs phenotype. Thus, LSC/CSCs may be transient states rather than entities. Recognizing adaptive phenotypic shift has great clinical importance. With LSC/CSC targeted therapies underway, the difference between treating an entity and a spectrum of dynamic reversible states will have conclusive effects on the outcome. The concept of APS may also be generalized to solid tumors in the contexts of dormancy, relapse, metastasis and cancer cell metabolism. The achievement of long-term clinical responses in targeted therapy will depend on successful targeting of adaptive phenotypic modulations in cancer cells.

## Supporting information

S1 Materials and Methods (DOCX)

S1 Results (DOCX)

S1 Raw image Original images. (PDF)

## References

[1] Quintás-Cardama A, Cortes J. Molecular biology of bcr-abl1-positive chronic myeloid leukemia. Blood. 2009;113: 1619–30. 10.1182/blood-2008-03-144790 18827185 PMC3952549

[2] Deininger MWN, Goldman JM, Melo JV, Dc W, Deininger MWN, Goldman JM, et al The molecular biology of chronic myeloid leukemia Review article The molecular biology of chronic myeloid leukemia. 2000;96: 3343–3356. 11071626

[3] Hochhaus A, Larson RA, Guilhot F, Radich JP, Branford S, Hughes TP, et al Long-Term Outcomes of Imatinib Treatment for Chronic Myeloid Leukemia. N Engl J Med. 2017;376: 917–927. 10.1056/NEJMoa1609324 28273028 PMC5901965

[4] Assouline S, Lipton JH. Monitoring response and resistance to treatment in chronic myeloid leukemia. Curr Oncol. 2011;18: e71–83. Available: http://www.ncbi.nlm.nih.gov/pubmed/21505592 21505592 10.3747/co.v18i2.391 PMC3070714

[5] Jabbour EJ, Cortes JE, Kantarjian HM. Resistance to tyrosine kinase inhibition therapy for chronic myelogenous leukemia: a clinical perspective and emerging treatment options. Clin Lymphoma Myeloma Leuk. 2013;13: 515–29. 10.1016/j.clml.2013.03.018 23890944 PMC4160831

[6] von Bubnoff N, Peschel C, Duyster J. Resistance of Philadelphia-chromosome positive leukemia towards the kinase inhibitor imatinib (STI571, Glivec): a targeted oncoprotein strikes back. Leukemia. 2003;17: 829–838. 10.1038/sj.leu.2402889 12750693

[7] Cipolleschi M, Rovida E, Sbarba P. The Culture-Repopulating Ability Assays and Incubation in Low Oxygen: A Simple Way to Test Drugs on Leukaemia Stem or Progenitor Cells. Curr Pharm Des. 2013;19: 5374–5383. 10.2174/1381612811319300006 23394087 PMC3821383

[8] Rovida E, Peppicelli S, Bono S, Bianchini F, Tusa I, Cheloni G, et al The metabolically-modulated stem cell niche: a dynamic scenario regulating cancer cell phenotype and resistance to therapy. Cell Cycle. 2014;13: 3169–3175. 10.4161/15384101.2014.964107 25485495 PMC4612663

[9] Kemper K, de Goeje PL, Peeper DS, van Amerongen R. Phenotype switching: tumor cell plasticity as a resistance mechanism and target for therapy. Cancer Res. 2014;74: 5937–41. 10.1158/0008-5472.CAN-14-1174 25320006

[10] Bhatia S, Monkman J, Kie A, Toh L, Nagaraj SH, Thompson EW. Targeting epithelial–mesenchymal plasticity in cancer: clinical and preclinical advances in therapy and monitoring. 2017; 3269–3306. 10.1042/BCJ20160782 28931648

[11] Prieto-García E, Díaz-García CV, García-Ruiz I, Agulló-Ortuño MT. Epithelial-to-mesenchymal transition in tumor progression. Med Oncol. 2017;34: 122 10.1007/s12032-017-0980-8 28560682

[12] Kalluri R, Weinberg RA. The basics of epithelial-mesenchymal transition. J Clin Invest. 2009;119: 1420–8. 10.1172/JCI39104 19487818 PMC2689101

[13] Cano A, Pérez-Moreno MA, Rodrigo I, Locascio A, Blanco MJ, del Barrio MG, et al The transcription factor Snail controls epithelial–mesenchymal transitions by repressing E-cadherin expression. Nat Cell Biol. 2000;2: 76–83. 10.1038/35000025 10655586

[14] Huber MA, Kraut N, Beug H. Molecular requirements for epithelial–mesenchymal transition during tumor progression. Curr Opin Cell Biol. 2005;17: 548–558. 10.1016/j.ceb.2005.08.001 16098727

[15] Deininger MWN, Druker BJ. Specific Targeted Therapy of Chronic Myelogenous Leukemia with Imatinib. Pharmacol Rev. 2003;55: 401–423. 10.1124/pr.55.3.4 12869662

[16] HIGHLIGHTS OF PRESCRIBING INFORMATION. [cited 4 Mar 2018]. http://www.iclusig.com/pi

[17] Deininger MWN, Druker BJ. Specific targeted therapy of chronic myelogenous leukemia with imatinib. Pharmacol Rev. 2003;55: 401–23. 10.1124/pr.55.3.4 12869662

[18] Deininger MW. Nilotinib. Clin Cancer Res. 2008;14: 4027–4031. 10.1158/1078-0432.CCR-07-5015 18593977

[19] Abbas R, Hug BA, Leister C, El Gaaloul M, Chalon S, Sonnichsen D. A phase I ascending single-dose study of the safety, tolerability, and pharmacokinetics of bosutinib (SKI-606) in healthy adult subjects. Cancer Chemother Pharmacol. 2012;69: 221–227. 10.1007/s00280-011-1688-7 21691746

[20] Takahashi N, Miura M, Scott SA, Niioka T, Sawada K. Pharmacokinetics of dasatinib for Philadelphia-positive acute lymphocytic leukemia with acquired T315I mutation. J Hematol Oncol. 2012;5: 23 10.1186/1756-8722-5-23 22587422 PMC3409074

[21] Acikgoz E, Guven U, Duzagac F, Uslu R, Kara M, Soner BC, et al Enhanced G2/M Arrest, Caspase Related Apoptosis and Reduced E-Cadherin Dependent Intercellular Adhesion by Trabectedin in Prostate Cancer Stem Cells. PLoS One. 2015;10: e0141090 10.1371/journal.pone.0141090 26485709 PMC4618065

[22] Zhang K, Yang G, Wu W, Zhang J, Xia X, Jiang T, et al Decreased Expression of Caveolin-1 and E-Cadherin Correlates with the Clinicopathologic Features of Gastric Cancer and the EMT Process. Recent Pat Anticancer Drug Discov. 2016;11: 236–44. Available: http://www.ncbi.nlm.nih.gov/pubmed/26817615 26817615 10.2174/1574892811666160128151437

[23] Liang W, Hao Z, Han J-L, Zhu D-J, Jin Z-F, Xie W-L. CAV-1 contributes to bladder cancer progression by inducing epithelial-to-mesenchymal transition. Urol Oncol Semin Orig Investig. 2014;32: 855–863. 10.1016/j.urolonc.2014.01.005 24968949

[24] Hwangbo C, Tae N, Lee S, Kim O, Park OK, Kim J, et al Syntenin regulates TGF-β1-induced Smad activation and the epithelial-to-mesenchymal transition by inhibiting caveolin-mediated TGF-β type I receptor internalization. Oncogene. 2016;35: 389–401. 10.1038/onc.2015.100 25893292

[25] Lu Z, Ghosh S, Wang Z, Hunter T. Downregulation of caveolin-1 function by EGF leads to the loss of E-cadherin, increased transcriptional activity of beta-catenin, and enhanced tumor cell invasion. Cancer Cell. 2003;4: 499–515. 10.1016/s1535-6108(03)00304-0 14706341

[26] Wong SHM, Fang CM, Chuah L-H, Leong CO, Ngai SC. E-cadherin: Its dysregulation in carcinogenesis and clinical implications. Crit Rev Oncol Hematol. 2018;121: 11–22. 10.1016/j.critrevonc.2017.11.010 29279096

[27] Jamieson CHM, Ailles LE, Dylla SJ, Muijtjens M, Jones C, Zehnder JL, et al Granulocyte–Macrophage Progenitors as Candidate Leukemic Stem Cells in Blast-Crisis CML. N Engl J Med. 2004;351: 657–667. 10.1056/NEJMoa040258 15306667

[28] Seton-Rogers S. Leukaemia Stem Cells: Homing in on CD44. Nat Rev Cancer. 2006;6: 832–832.

[29] Jin L, Hope KJ, Zhai Q, Smadja-Joffe F, Dick JE. Targeting of CD44 eradicates human acute myeloid leukemic stem cells. Nat Med. 2006;12: 1167–1174. 10.1038/nm1483 16998484

[30] Hertweck MK, Erdfelder F, Kreuzer K, Hertweck MK, Erdfelder F, Cd KK, et al CD44 in hematological neoplasias To cite this version: HAL Id: hal-00610424 CD44 in hematological neoplasias. 2011;90: 493–508. 10.1007/s00277-011-1161-z 21258793

[31] Krause DS, Lazarides K, Von Andrian UH, Van Etten RA. Requirement for CD44 in homing and engraftment of BCR-ABL-expressing leukemic stem cells. Nat Med. 2006;12: 1175–1180. 10.1038/nm1489 16998483

[32] Holyoake TL, Vetrie D. The chronic myeloid leukemia stem cell: stemming the tide of persistence. 2017;129: 1595–1607. 10.1182/blood-2016-09-696013 28159740

[33] Corbin AS, Agarwal A, Loriaux M, Cortes J, Deininger MW, Druker BJ. Human chronic myeloid leukemia stem cells are insensitive to imatinib despite inhibition of BCR-ABL activity. J Clin Invest. 2011;121: 396–409. 10.1172/JCI35721 21157039 PMC3007128

[34] Ishiguro T, Ohata H, Sato A, Yamawaki K, Enomoto T, Okamoto K, et al Tumor-derived spheroids: Relevance to cancer stem cells and clinical applications. Cancer Sci. 2017;108: 283–289. 10.1111/cas.13155 28064442 PMC5378268

[35] Xin H, Kong Y, Jiang X, Wang K, Qin X, Miao ZH, et al Multi-drug-resistant cells enriched from chronic myeloid leukemia cells by doxorubicin possess tumor-initiating-cell properties. J Pharmacol Sci. 2013;122: 299–304. 10.1254/jphs.13025fp 23903006

[36] Mandl EW, Jahr H, Koevoet JLM, Van Leeuwen JPTM, Weinans H, Verhaar JAN, et al Fibroblast growth factor-2 in serum-free medium is a potent mitogen and reduces dedifferentiation of human ear chondrocytes in monolayer culture. Matrix Biol. 2004;23: 231–241. 10.1016/j.matbio.2004.06.004 15296937

[37] Matsuo T, Takabatake M, Matsuo N. The Effects of Growth Factors on Multicellular Spheroids Formed by Chick Embryonic Retinal Cells. Acta Med Okayama. 1997;51: 251–260. 10.18926/AMO/30788 9359922

[38] Lee J, Lee JY, Chae BC, Jang J, Lee EA, Son Y. Fully Dedifferentiated Chondrocytes Expanded in Specific Mesenchymal Stem Cell Growth Medium with FGF2 Obtains Mesenchymal Stem Cell Phenotype In Vitro but Retains Chondrocyte Phenotype In Vivo. Cell Transplant. 2017;26: 1673–1687. 10.1177/0963689717724794 29251111 PMC5753982

[39] Puissant A, Dufies M, Fenouille N, Ben Sahra I, Jacquel A, Robert G, et al Imatinib triggers mesenchymal-like conversion of CML cells associated with increased aggressiveness. J Mol Cell Biol. 2012;4: 207–220. 10.1093/jmcb/mjs010 22467682

[40] Staal FJT, Luis TC. Wnt signaling in hematopoiesis: Crucial factors for self-renewal, proliferation, and cell fate decisions. J Cell Biochem. 2010;109: n/a–n/a. 10.1002/jcb.22467 20069555

[41] Jolly MK, Mani SA, Levine H. Hybrid epithelial/mesenchymal phenotype(s): The ‘fittest’ for metastasis? Biochim Biophys Acta—Rev Cancer. 2018;1870: 151–157. 10.1016/j.bbcan.2018.07.001 29997040

[42] Zöller M. CD44, hyaluronan, the hematopoietic stem cell, and leukemia-initiating cells. Front Immunol. 2015;6: 1–23. 26074915 10.3389/fimmu.2015.00235 PMC4443741

[43] Tetteroo PAT, Massaro F, Mulder A, Schreuder-van Gelder R, von dem Borne AEGK. Megakaryoblastic differentiation of proerythroblastic K562 cell-line cells. Leuk Res. 1984;8: 197–206. 10.1016/0145-2126(84)90143-7 6232432

[44] Zanyk MJ, Banerjee D, McFarlane DL. Flow cytometric analysis of the phenotypic changes in tumour cell lines following TPA induction. Cytometry. 1988;9: 374–379. 10.1002/cyto.990090415 3402283

[45] ANDERSSON LC, JOKINEN M, GAHMBERG CG. Induction of erythroid differentiation in the human leukaemia cell line K562. Nature. 1979;278: 364–365. 10.1038/278364a0 570644

[46] Gambari R, del Senno L, Barbieri R, Viola L, Tripodi M, Raschellà G, et al Human leukemia K-562 cells: induction of erythroid differentiation by 5-azacytidine. Cell Differ. 1984;14: 87–97. 10.1016/0045-6039(84)90033-2 6205767

[47] Bianchi Scarrà GL, Romani M, Coviello DA, Garrè C, Ravazzolo R, Vidali G, et al Terminal erythroid differentiation in the K-562 cell line by 1-beta-D-arabinofuranosylcytosine: accompaniment by c-myc messenger RNA decrease. Cancer Res. 1986;46: 6327–32. Available: http://www.ncbi.nlm.nih.gov/pubmed/3536078 3536078

[48] Tonini GP, Radzioch D, Gronberg A, Clayton M, Blasi E, Benetton G, et al Erythroid differentiation and modulation of c-myc expression induced by antineoplastic drugs in the human leukemic cell line K562. Cancer Res. 1987;47: 4544–7. Available: http://www.ncbi.nlm.nih.gov/pubmed/3476195 3476195

[49] Ren J-G, Seth P, Everett P, Clish CB, Sukhatme VP. Induction of erythroid differentiation in human erythroleukemia cells by depletion of malic enzyme 2. PLoS One. 2010;5 10.1371/journal.pone.0012520 20824065 PMC2932743

[50] Chorzalska A, Kim JF, Roder K, Tepper A, Ahsan N, Rao RSP, et al Long-Term Exposure to Imatinib Mesylate Downregulates Hippo Pathway and Activates YAP in a Model of Chronic Myelogenous Leukemia. Stem Cells Dev. 2017;26: 656–677. 10.1089/scd.2016.0262 28103766 PMC5421616

[51] Hochedlinger K, Plath K. Epigenetic reprogramming and induced pluripotency. Development. 2009;136: 509–523. 10.1242/dev.020867 19168672 PMC2685952

[52] Surani MA, Hayashi K, Hajkova P. Genetic and Epigenetic Regulators of Pluripotency. Cell. 2007;128: 747–762. 10.1016/j.cell.2007.02.010 17320511

