# Supplementary material for: Adaptive phenotypic modulations lead to therapy resistance in chronic myeloid leukemia cells
Source: PLoS One. 2020 Feb 27;15(2):e0229104. doi: 10.1371/journal.pone.0229104 (PMC7046262; doi:10.1371/journal.pone.0229104)
Supplement: S1 Materials and Methods — (DOCX) [file pone.0229104.s001.docx]

**Supporting Materials and Methods**

*Antibodies used in Western blot Analysis*

anti-CD44 (R&D #SC017 kit), anti-Caveolin (sc-894), anti-ß-catenin (Cell Signaling #8480), PathScan® Bcr-Abl Activity Assay: Multiplex Western Detection Cocktail (Cell Signaling #7130), PathScan® PDGFR Activity Assay: Multiplex Western Detection Cocktail II (Cell Signaling #5304)

*Flow cytometry assay for surface molecules*

Cells in suspension were collected by centrifugation and fixed with 3.7% formaldehyde for 15 min at room temperature. After washing steps with PBS, cells were stained with the conjugated-primary antibody or native primary antibody for 1h at room temperature in %5 BSA/PBS buffer. After washing steps with PBS, cells were stained with conjugated-secondary antibody for 30 min at room temperature in %5 BSA/PBS buffer. After washing steps, cells are re-suspended in 500μl PBS and analyzed with BD FACS Calibur. Positive staining for each marker was determined by comparison with the only secondary antibody or appropriate isotype-matched of conjugated-primary antibodies as controls. Antibodies used for Cluster Differentiation (CD) molecules are against; CD19-PE (BD Bioscience 345777), CD44 (R&D SC017), CD45 (R&D SC017) , CD90 (R&D SC017), CD105 (R&D SC017), CD106 (R&D SC017), CD146 (R&D SC017), CD166 (R&D SC017), Stro-1 (R&D SC017), CD3 (BD Biosciences 332771), CD10 (BD Biosciences 332776), CD13 (BD Biosciences 347406), CD15 (BD Bioscience 561716), CD19 (BD Biosciences 345791), CD22 (BD Pharmingen 555425), CD24 (BD Pharmingen 555428), CD33 (BD Biosciences 345800), CD34 (BD Biosciences 348053), CD38 (BD Biosciences 345806), CD45 (R&D SC017; BD Biosciences 345809), CD62E (BD Pharmingen 551145), CD65 (eBioscience Inc. 11-0659-41), CD117 (BD Biosciences 332785), CD123 (BD Pharmingen 561009)

*Genome-wide gene expression data analysis*

The scanned data were first analyzed using Illumina GenomeStudio. Raw data were background corrected and exported for further analysis. Quantile normalization and variance-stabilizing transformation of background corrected data was performed using Lumi (Du, Kibbe and Lin, 2008) package in R (version 3.0.2, The R Foundation for Statistical Computing, Vienna, Austria; http://www.r-project.org). After pre-processing, only probes that passed the detection filter (detection p-value < 0.01) in one of the three replicates retained in the data. The annotations of the detected Illumina probes were derived using the nuID determined by Lumi (Du, Kibbe and Lin, 2008). Differentially expressed genes between groups (S vs. K; IR w/ IM vs. K; S vs. IR w/ IM; IR w/o IM vs. K; IR w/o IM vs. IR w/ i) were identified using LIMMA (Linear Models for Microarray Data) package (Smyth, 2004). A gene was called differentially expressed by LIMMA when its FDR-adjusted p-value was less than 0.05. Differentially expressed genes were further filtered by fold-change. The up-regulated and down-regulated genes with a ratio above a threshold for significantly higher (≥ 2 fold change) and lower expression (≤ 2 fold change) intensity were reported as differentially expressed genes. Differentially expressed genes between groups were divided into functionally relevant groups according DAVID Bioinformatics Resources (<http://david.abcc.ncifcrf.gov>) version 6.7.

*Quantitative real-time PCR for microarray validations*

RNA samples were extracted using Roche High Pure RNA isolation kit (Cat no: 11 828 665 001). RNAs were reverse transcripted using Fermentas First Strand cDNA synthesis kit #K1612. Q-PCR was performed using Roche Light Cycler Syber Green FastStart kit (# 12 239 264 001) or Thermo Scientific DyNAmo Flash Syber Green qPCR Kit (#F-415L). The primers were designed using the web-based "IDT Oligodesign" program (www.idtdna.com) to be compatible with conventional PCR and real-time PCR. Analyses were done with Roche Capillaire LightCycler v.4.0.0.23 software (Roche Diagnostics, Mannheim, Germany). B-actin or GAPDH gene primers were used for internal control. Every target gene primer was optimized separately and analyzed three times in triplicate. Q-PCR products lengths were checked by agarose gel electrophoresis (1%-1.2% gel). Primers used in Q-PCR analyses are listed in S3 Table.

*Transdifferentiation Experiments*

*Adipose tissue cell differentiation procedure*

Protocol was tested on the 3T3L1 (ATCC® CL-173™) cell line before the K562 cell lines. *Induction medium:* DMEM High glucose with 0.5mM isobutylmethylxanthine (IBMX), 1μM Dexamethasone, 10ug/ml insulin %10 FBS %1 L-Glutamine %1 P/S. *Insulin medium:* DMEM high glucose with10µg/ml insulin, %10 FBS, %1 L-glutamine, %1 P/S. Induction and insulin medium were prepared fresh before use.

Cells were seeded in a six-well plate at a density of 1x10^5^ cells. Cells were cultured in DMEM until confluency of 70% is reached, then the medium was changed every 2–3 days. To initiate differentiation, DMEM was removed and 2ml induction medium was added to each well (Day 0). On day 3, the induction medium was removed and replaced with 2ml insulin medium. On day 6, insulin medium was removed from the cells and replaced with induction medium. This cycle was performed until day 15. On the day 15, differentiation into adipocyte-like cells was tracked by Oil Red O staining to monitor lipid accumulation (Supporting Results Figure S3A). The same procedures were done by K562, K562-IR w/o IM, and K562-IR w/ IM cells (Supporting Results, S3B Figure). Experiments were done at three different time points.

*Bone tissue cell differentiation procedure*

Millipore Mesenchymal Stem Cell Osteogenesis Kit #SCR028 was used for the experiments according to the manufacturer's protocol. On day 15, differentiation was tracked by Alizarin Red staining. The assay results cannot be interpreted because of strong positive staining in both cell lines. K562 and K562-IR cells were analyzed for osteocalcin osteocyte marker by flow cytometry at a different time point instead of Alizarin red staining (Supporting Results, S3C Figure). R&D Human Osteocalcin PE-conjugated Antibody Monoclonal Mouse IgG1Clone #190125, Mouse IgG1 Isotype Control-PE, antibodies were used.

**S1 Table**

| NCBI Reference sequence:  NM_005157.4 |  | Sequences 5’ 3’ | Annealing  temperature |
| --- | --- | --- | --- |
| Primer 1 | Forward | ACAAGCCCACTGTCTATGGTGTGT | 58°C |
|  | Reverse | AGATCTGAGTGGCCATGTACAGCA |  |
| Primer 2 | Forward | ACAGAGATCTTGCTGCCCGAAACT | 58°C |
|  | Reverse | TCAGAGGGATTCCACTGCCAACAT |  |
| Primer 3 | Forward | TGAAGACCTTGAAGGAGGACACCA | 58°C |
|  | Reverse | AGCAATACTCCAAATGCCCAGACG |  |
| Primer 4 | Forward | ACGTCTGGGCATTTGGAGTATTGC | 58°C |
|  | Reverse | CAAGGTACTCACAGCCCCACGGAC |  |

**S1 Table:** The primer sequences used to generate four overlapping fragments in the Bcr-Abl kinase domain. These primers were used to screen for M244V, L248R, L248V, G250E, Q252H, Y253F, Y253H, E255K, E255V, D276G, E279K, E292L, V299L, T315A, T315I, T315V, F317L, F317R, F317V, M343T, M351T, F359I, F359V, L384M, H396P, H396R, and F486S BCR-Abl kinase domain mutations.

**S2 Table**

| Primer name | Sequence 5’🡪3’ |
| --- | --- |
| Major-minor BCR-ABL BCR forward | TCCGCTGACCATCAATAAGGA |
| Major-minor BCR-ABL ABL reverse | CACTCAGACCCTGAGGCTCAA |
| BCR-ABL hydrolysis probe | CCCTTCAGCGGCCAGTAGCATCTGA |

**S2 Table:** The primer sequences used in the probe-based BCR-Abl quantitative PCR analysis. Annealing temperature is 58°C.

**S3 Table**

| Gene name | NCBI Referance sequence | Forward 5’🡪3’  Reverse 5’ 🡪3’ | Annealing degree |
| --- | --- | --- | --- |
| EMP1 | NM_001423.1 | F: TTGCCCTCCTGGTCTTCGTG  R: TAGCCGTGGTGATACTGCGTT | 62°C |
| S100A10 | NM_002966.2 | F: TGTAGAGATGGCAAAGTGGG  R: TTTATTGAGGGCAAGGGGATG | 57°C |
| BMP6 | NM_001718.4 | F: ACTCTGACCTGTTTTTGTTGGA  R:TGTTGTAATCTGAAGCACTGGA | 58°C |
| IGFBP5 | NM_000599.2 | F: TTCCACCCATTCTCCCTTTG  R: GTAGTTCCTGGCTCAGTCTTT | 57°C |
| PBX1 | NM_002585.1 | F:CCAGTGAGGAAGCCAAAGAGGA  R: TGGGAGTTGAGGGCGAGTTAG | 62°C |
| SRGN | NM_002727.2 | F:ACTTGAATCGTATCTTCCCACTTT  R:GTAATCCTGTTCCATTTCCGTTAG | 58°C |
| BTG2 | NM_006763.2 | F: CTCCTCTAACCCTCCCCTCC  R: GACTAGCCAGCCATCATCCC | 60°C |
| PIM1 | NM_002648.2 | F: CTCCTCTGACTTGGGGACCT  R: GGCTACCTGCTGCTCAAAAC | 60°C |

**S3 Table:** The primer sequences used in the Q-RT-PCR analyses for the genome-wide mRNA microarray gene expression validations
