# Supplementary material for: Adaptive phenotypic modulations lead to therapy resistance in chronic myeloid leukemia cells
Source: PLoS One. 2020 Feb 27;15(2):e0229104. doi: 10.1371/journal.pone.0229104 (PMC7046262; doi:10.1371/journal.pone.0229104)
Supplement: S1 Results — (DOCX) [file pone.0229104.s002.docx]

**Supporting Results**

**S1 Figure**

**A.**

**A.**

**
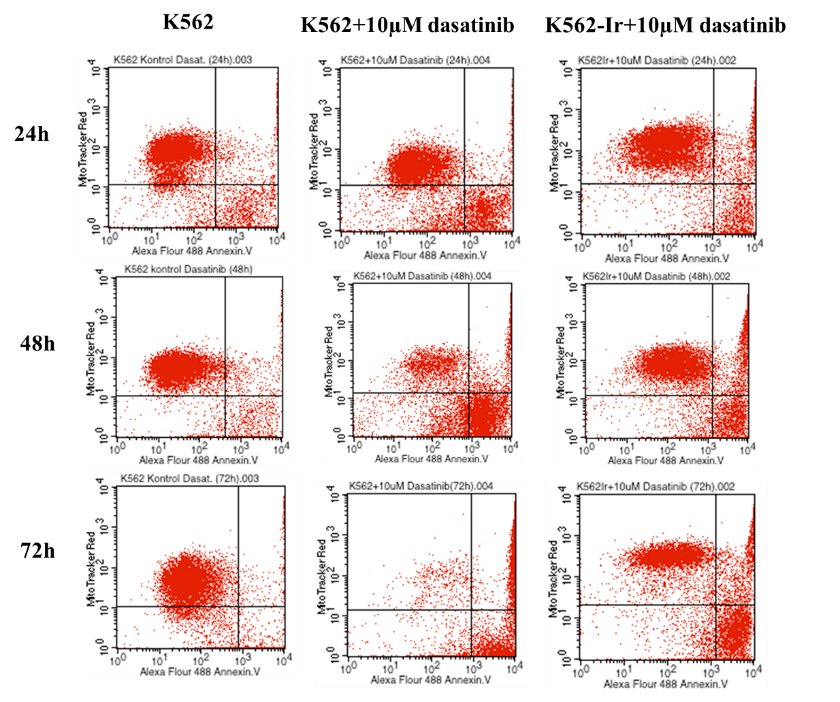

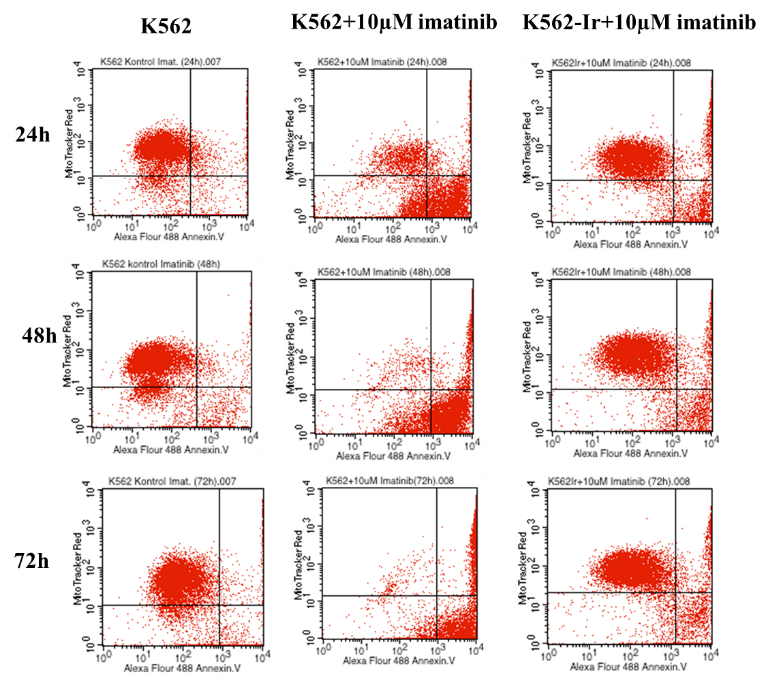
**

**B.**

**
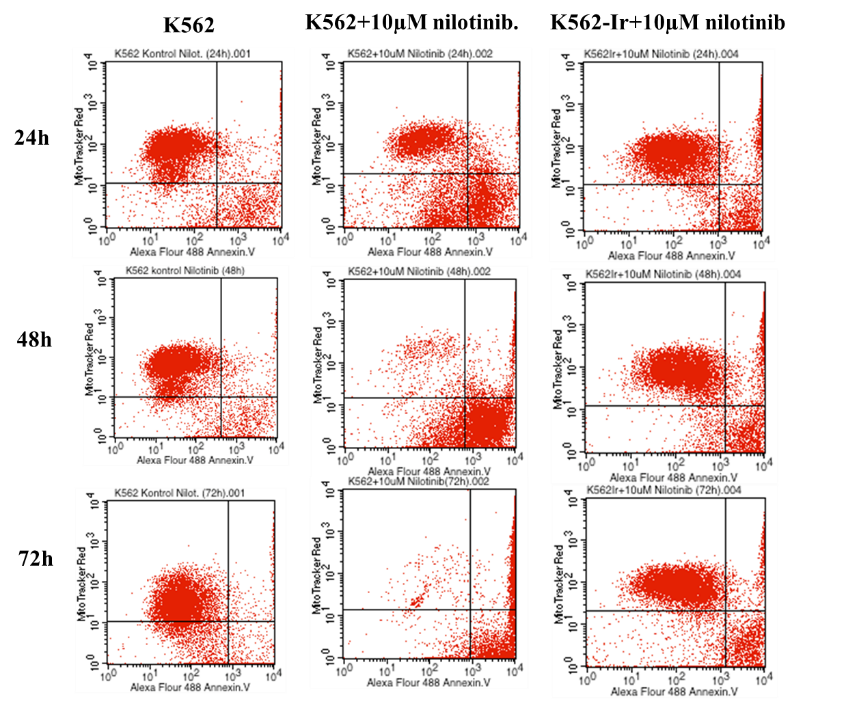

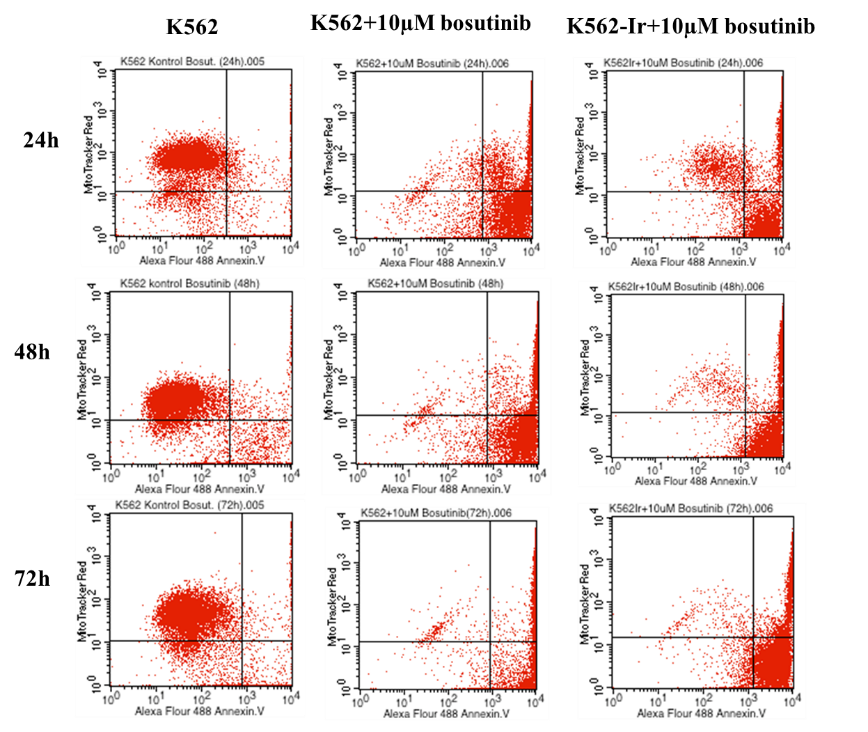
**

**C.**

**D.**

**E.**


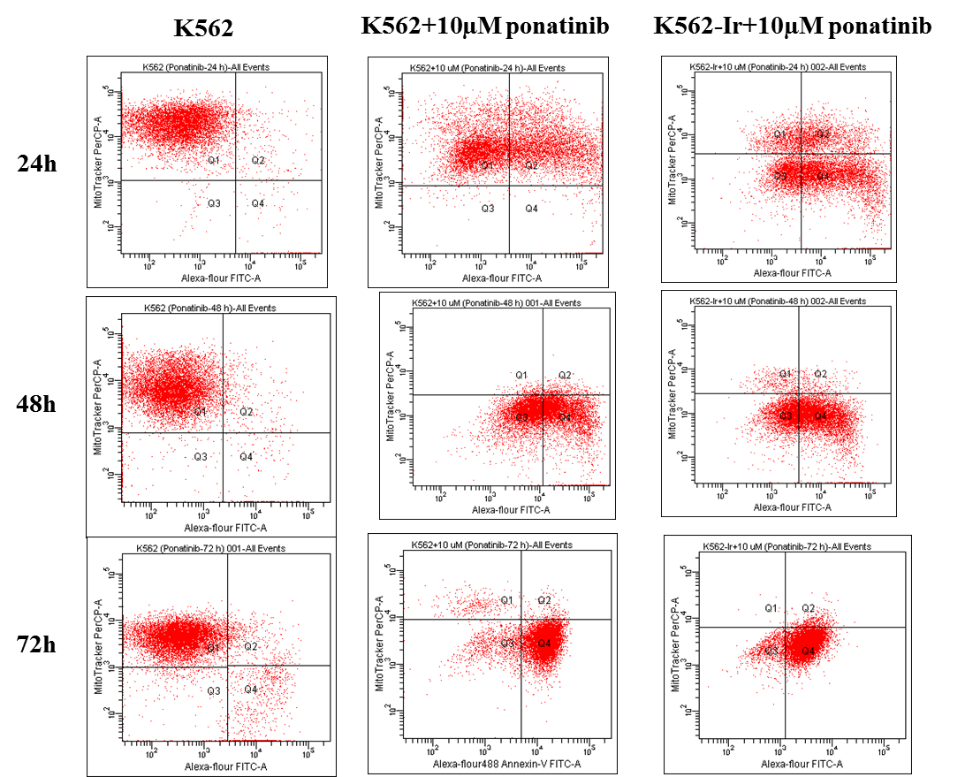


**S1 Fig.:** Flow cytometry results of 1^st^, 2^nd^ and 3^rd^ generation TKI resistance observed in K562-IR. Mitotracker Red/PI analyses done after 24, 48 and 72h TKI treatment of K562 cells, K562 cells with 10µM imatinib added 24h prior experiment and K562-IR cells grown in media with 10µM imatinib. **A.** imatinib treatment **B.** dasatinib treatment. **C.** nilotinib treatment **D.** bosutinib treatment **E.** ponatinib treatment.

**Figure S2**

| # | sample | parental/reference | match/comment |
| --- | --- | --- | --- |
| 1 | K562 | K-562 (DSMZ ACC 010) | full-matching STR profile, authentic |
| 2 | K562-IR | K-562 (DSMZ ACC 010) | full-matching STR profile, authentic |

**S2A Fig.:** Authentication analysis results. Authentication analyses of the samples were done by Leibniz-Institut DSMZ GmbH (Germany).

**
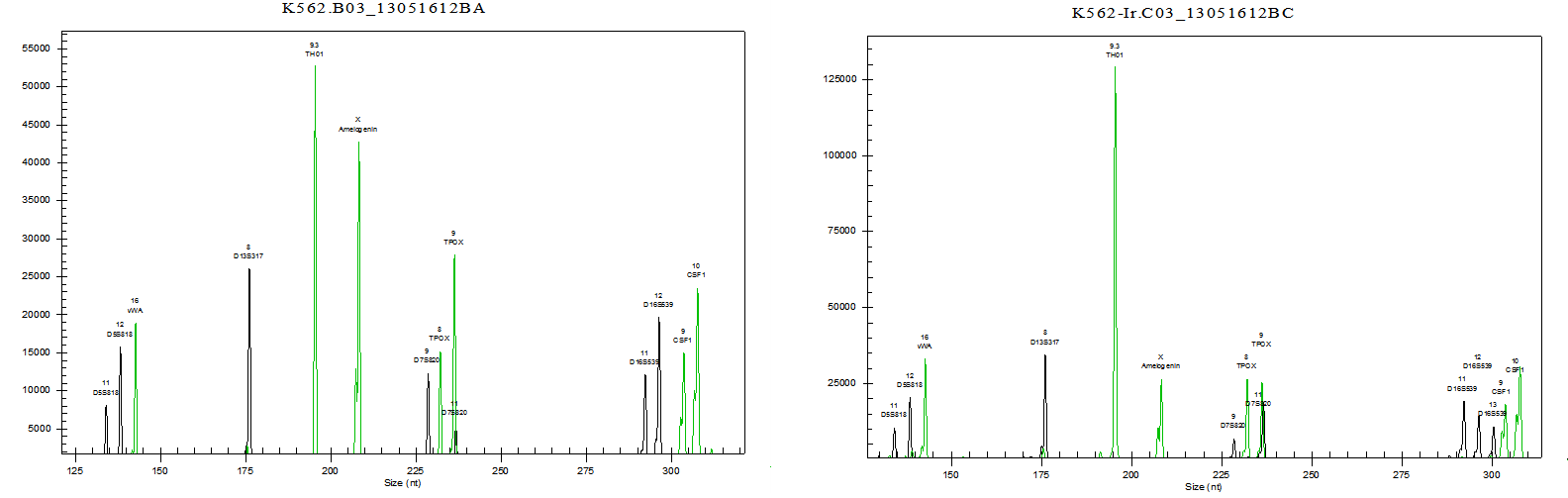
**

**S2B Fig.:** Electropherograms of the samples.

**S2C Fig.:** Allelic list of STR-profile of the samples

**S3Figure**

**
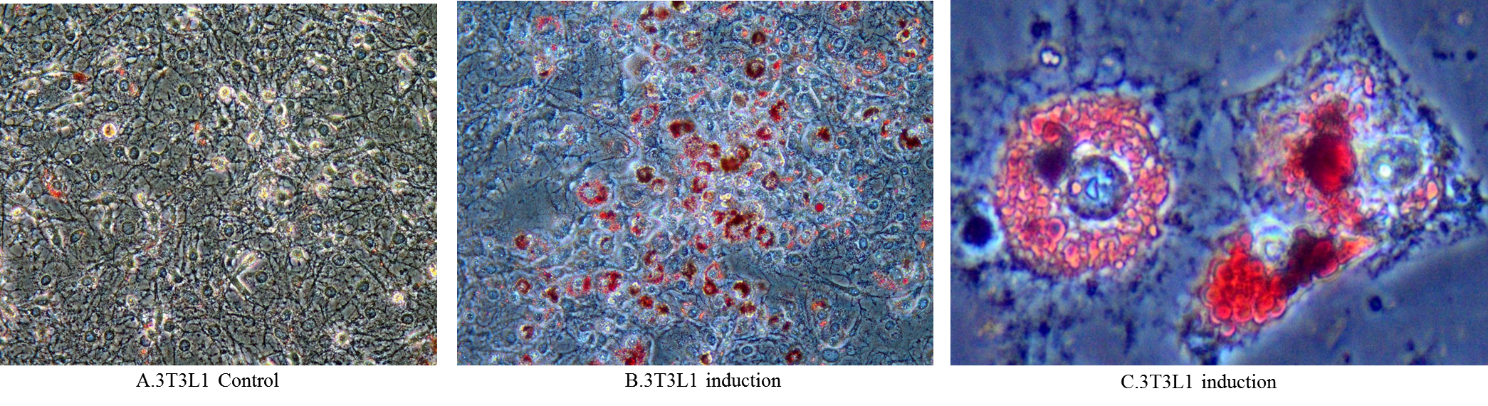
**

**S3A
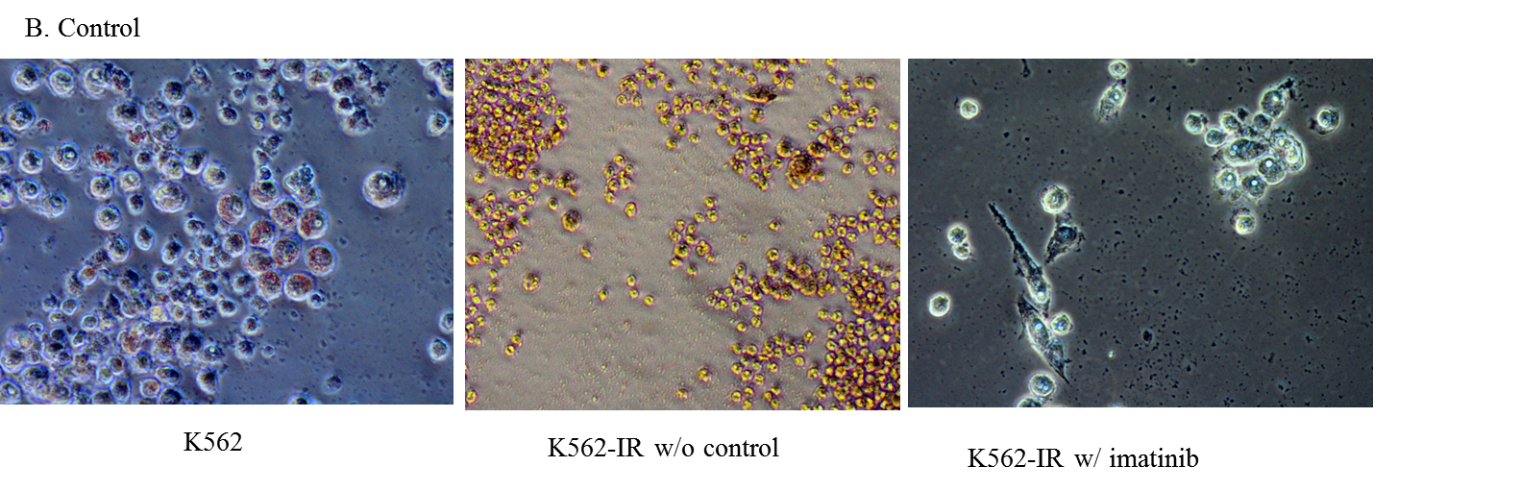

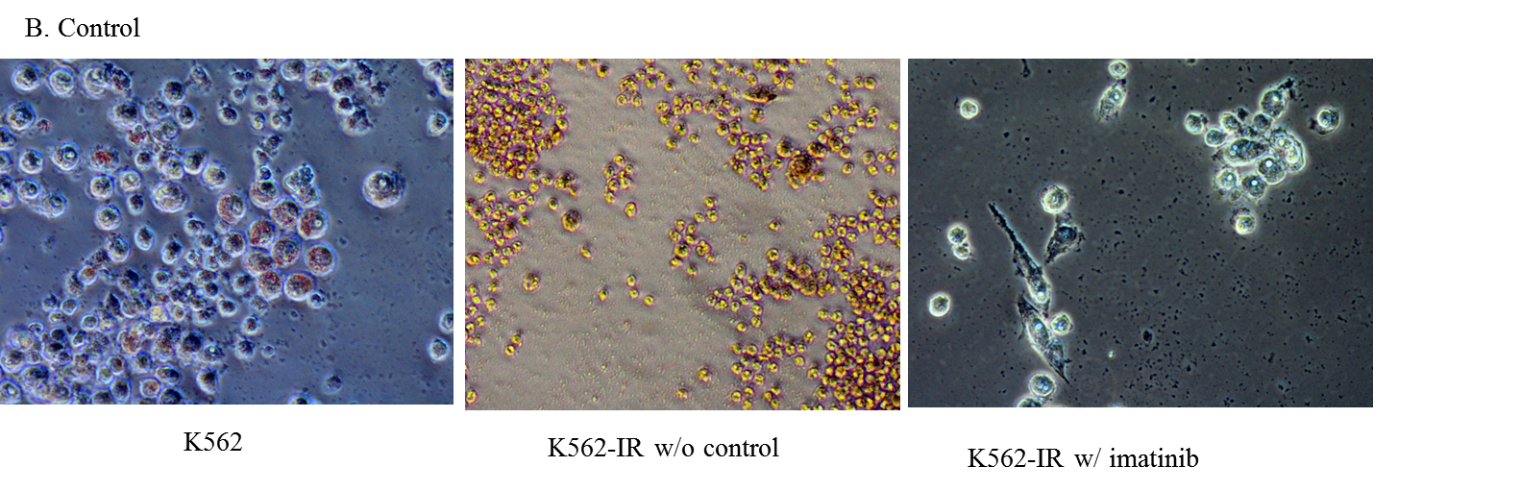

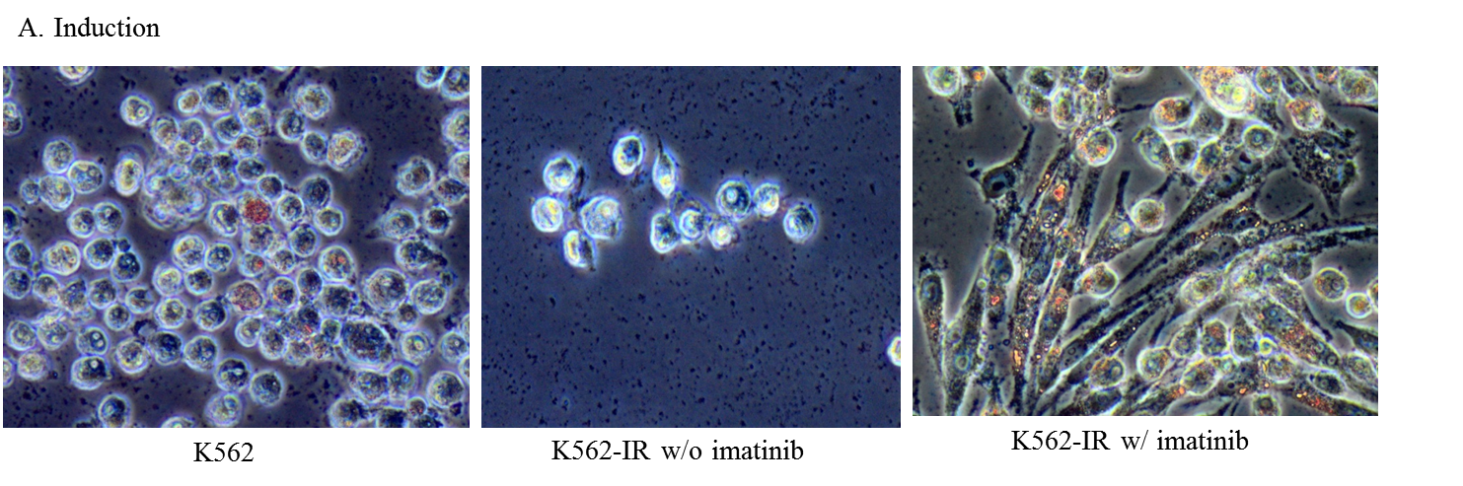

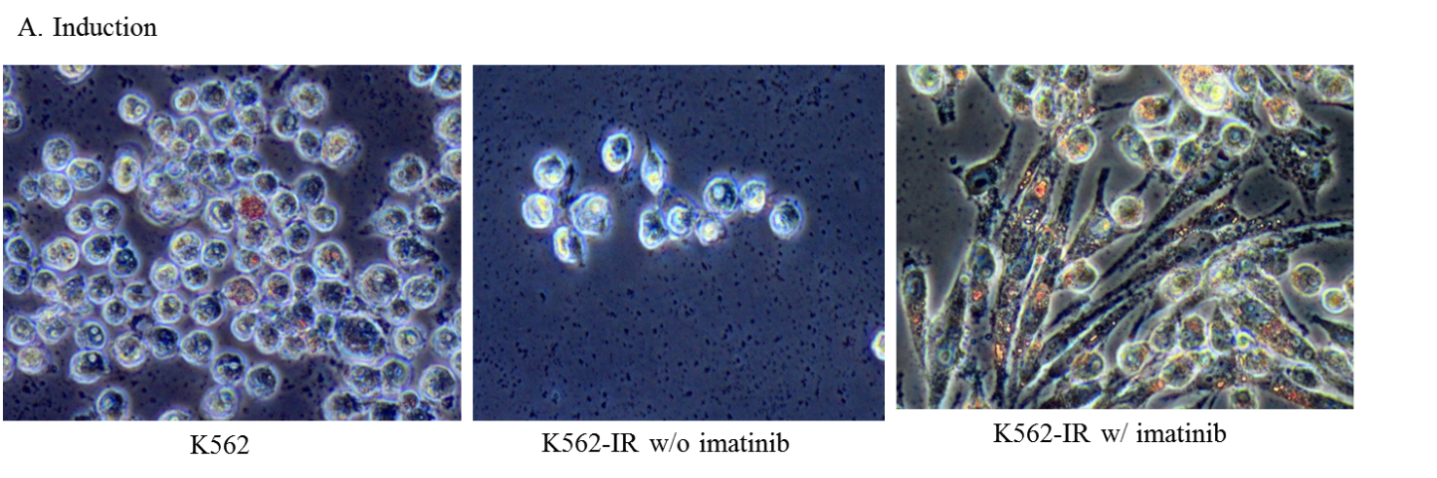
Fig.:** 3T3L1 adipocyte differentiation. **A.** 3T3L1 cell photos of control wells. **B.** 3T3L1 cells photos after 15 days of induction. Rounded differentiated cells are seen. **C.** Magnified adipocyte cells with triglycerides droplets.

**S3B Fig.:** K562 and K562-IR cells adipocyte differentiation. A. K562, K562-Ir w/o imatinib and K562-IR w/ imatinib induction well photos on day 15. B. K562, K562-Ir w/o imatinib and K562-IR w/ imatinib control well photos on day 15.


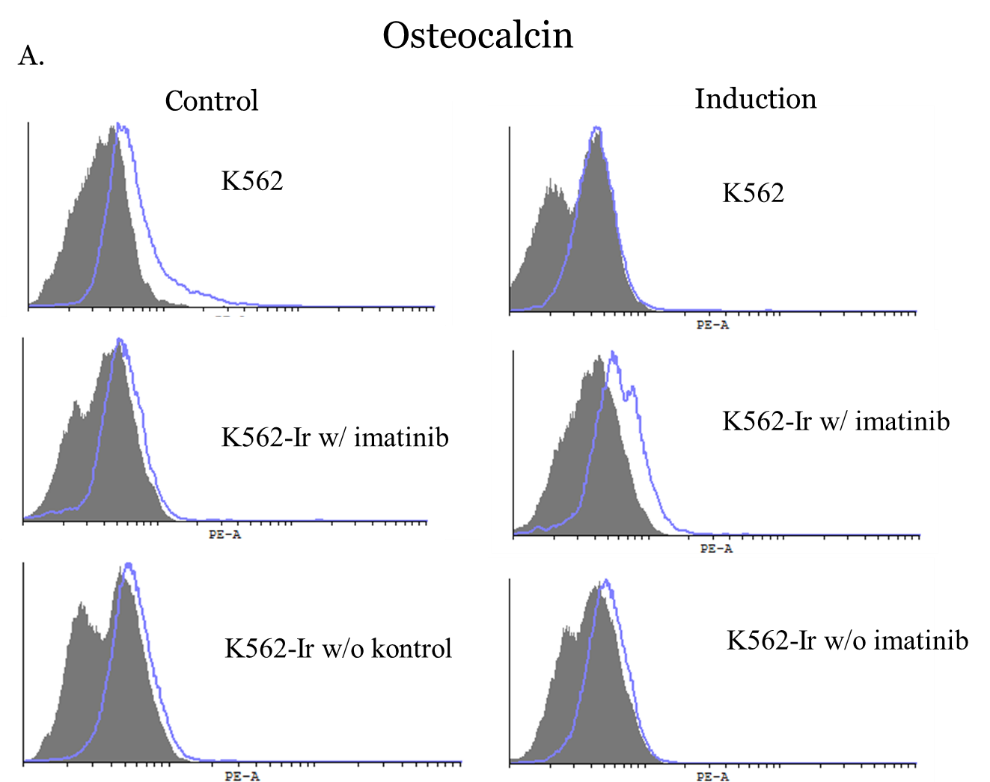


[Belgeden yaptığınız güzel bir alıntıyla okurlarınızın dikkatini çekin veya önemli bir noktayı vurgulamak için bu alanı kullanın. Bu metin kutusunu sayfada herhangi bir yere yerleştirmek için sürüklemeniz yeterlidir.]

**S3C Fig.:** Flow cytometry analyses of K562 and K562-IR cells for osteocalcin marker. Grey area in the histogram is background signal from isotype control.

**S4 Figure**

**
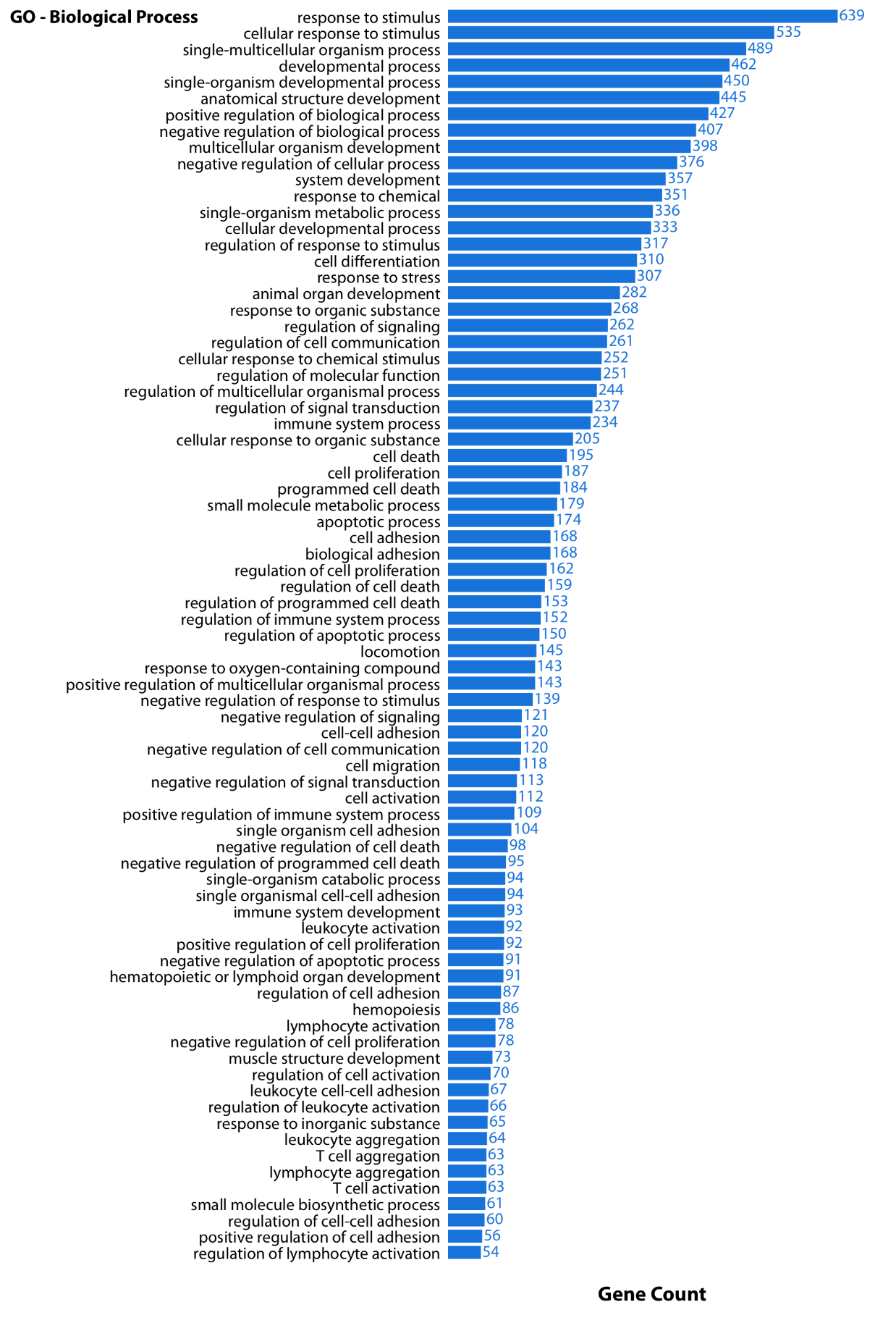
**

**S4 Fig.:** Gene ontology biological process enrichment analysis of IR w/ IM vs. K DEG list. The bar plot represents the number of differentially expressed genes that are annotated with each GO term (FDR<0.05). Only GO biological process terms that have FDR-adjusted enrichment p-values < 0.05 were included.

[Belgeden yaptığınız güzel bir alıntıyla okurlarınızın dikkatini çekin veya önemli bir noktayı vurgulamak için bu alanı kullanın. Bu metin kutusunu sayfada herhangi bir yere yerleştirmek için sürüklemeniz yeterlidir.]

**S5 Figure**

**
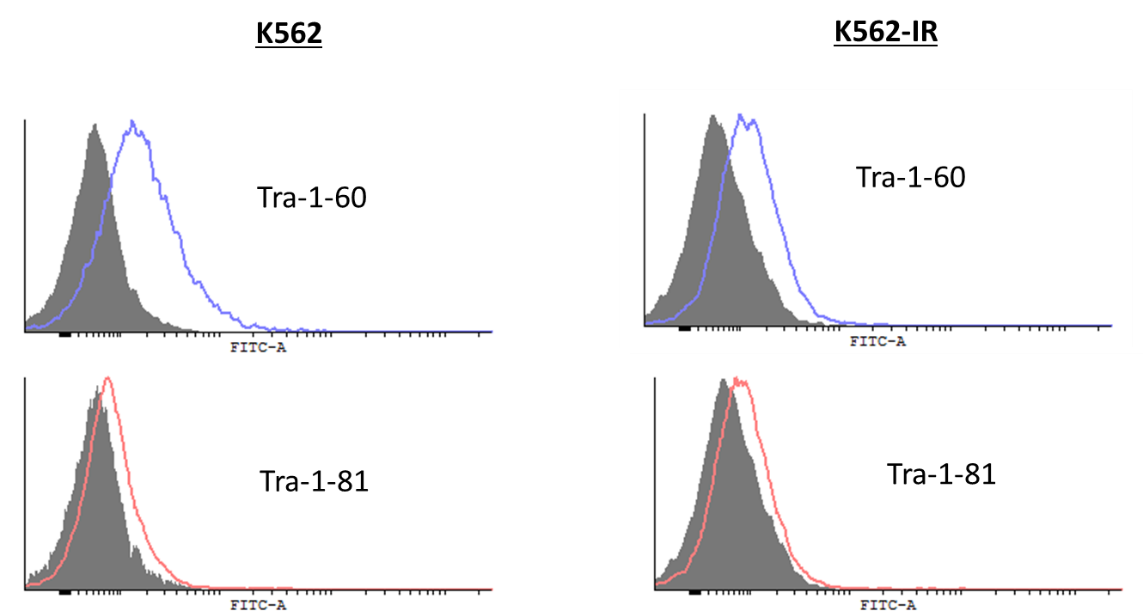
**

**S5 Fig.:** Flow cytometry analysis of Tra-1-60 and Tra-1-81 cell surface markers. Grey areas are isotype controls.

**
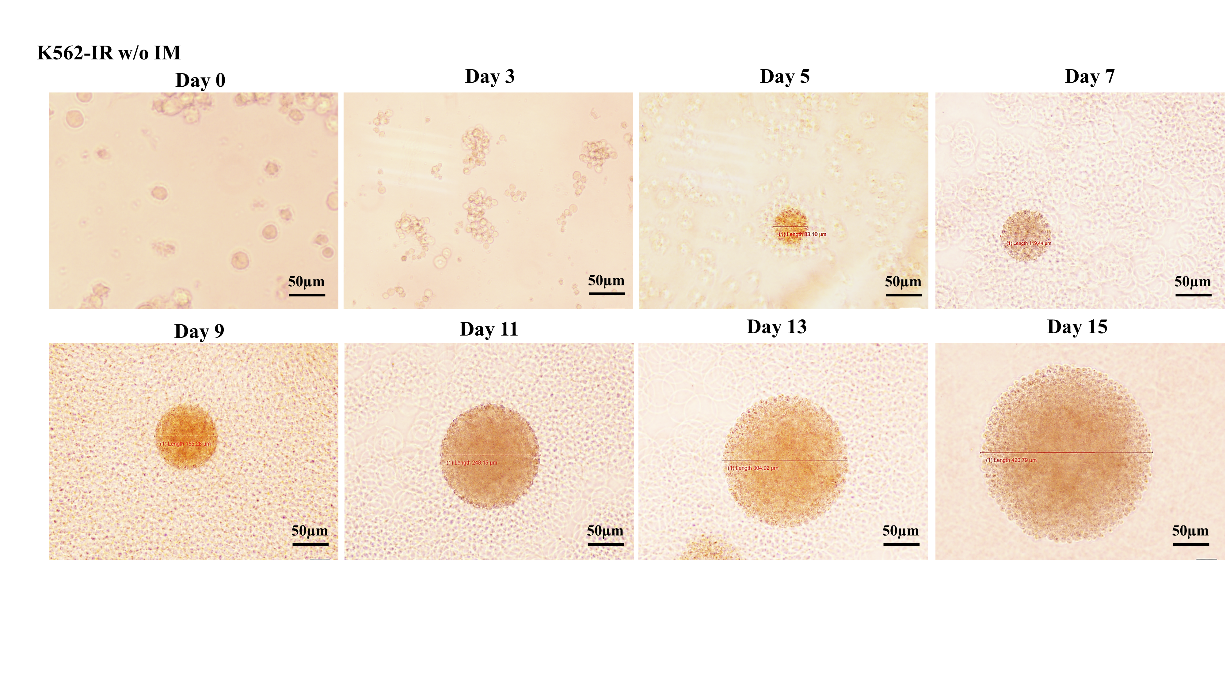
S6 Figure**

**S6 Figure:** Spheroid formation assay of K562 and K562-IR w/o IM cells. K562-IR cells were cultured without imatinib as K562 cells spheroid assay conditions.
