# Supplementary material for: Adaptive phenotypic modulations lead to therapy resistance in chronic myeloid leukemia cells
Source: PLoS One. 2020 Feb 27;15(2):e0229104. doi: 10.1371/journal.pone.0229104 (PMC7046262; doi:10.1371/journal.pone.0229104)
Supplement: S1 Raw image — (PDF) [file pone.0229104.s003.pdf]

Orginal images

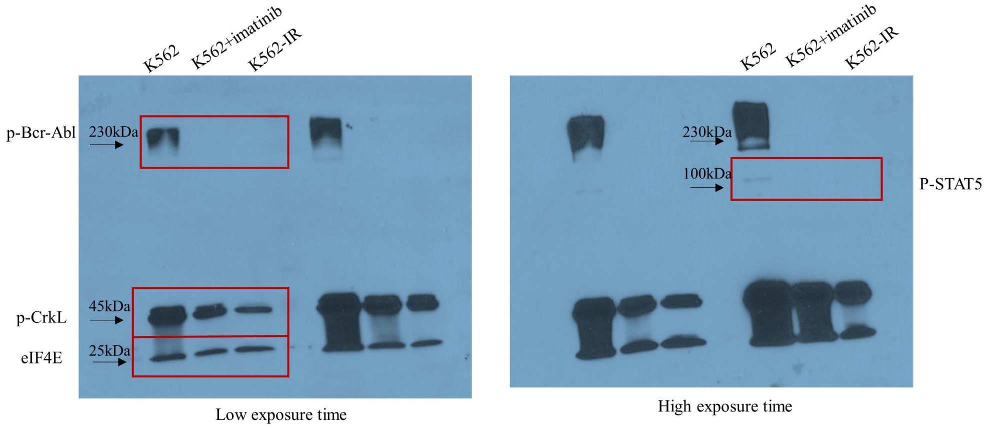

BCR-Abl Western blot original gel image. Here we used a cocktail antibody (Cell Signaling #7130) containing four different antibodies; p-Bcr-Abl, p-STAT5, p-CrkL and eIF4E. Samples are studied in duplicate. Red-marked areas are used in the article. Because of the p-STAT5 band is weak, two separate exposure times are used and the image visualized separately.The figure on the left is obtained with low exposure and the figure on the right is obtained with high exposure. X-ray films are used for detection and imaging.

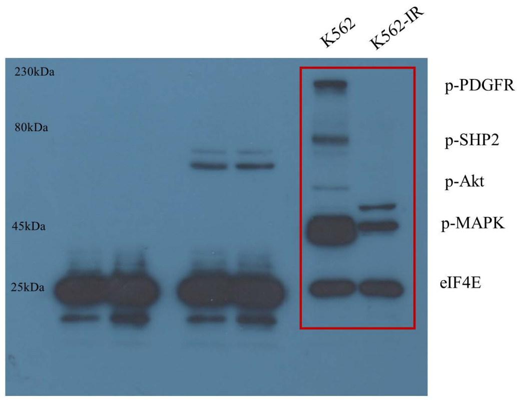

PDGFR Western blot original gel image. Here we used a cocktail antibody (Cell Signaling #5304) containing five different antibodies; p-PDGFR, p-SHP2, p-Akt p-MAPK and eIF4E. Red-marked area is used in the article, other lanes are irrelevant. X-ray films are used for detection and imaging.

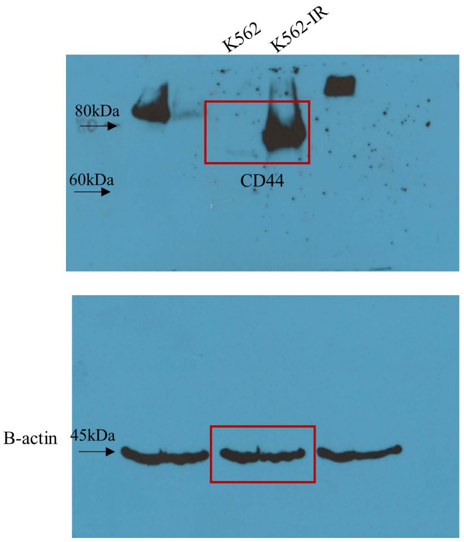

CD44 Western blot original gel image. Red-marked areas are used in the article. The lanes on the left and the right side of the gel are irrelevant. B-actin is used for loading control after mild-stripping. X-ray films are used for detection and imaging

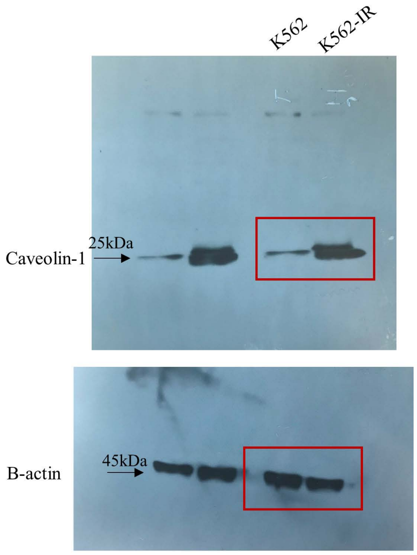

Caveolin-1 Western blot original gel image. Red-marked areas are used in the article. The lanes on the left side of the gel are duplicates. B-actin is used for loading control after mild-stripping. X-ray films are used for detection and imaging

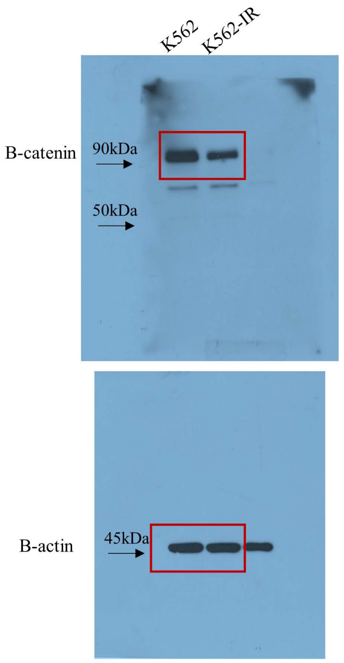

B-catenin Western blot original gel image. Red-marked areas are used in the article. The third lane on the right is irrevelant. B-actin is used for loading control after mild-stripping. X-ray films are used for detection and imaging.
